# Determining the Quantitative Principles of T Cell Response to Antigenic Disparity in Stem Cell Transplantation

**DOI:** 10.3389/fimmu.2018.02284

**Published:** 2018-10-11

**Authors:** Ali Salman, Vishal Koparde, Charles E. Hall, Max Jameson-Lee, Catherine Roberts, Myrna Serrano, Badar AbdulRazzaq, Jeremy Meier, Caleb Kennedy, Masoud H. Manjili, Stephen R. Spellman, Dayanjan Wijesinghe, Shahrukh Hashmi, Greg Buck, Rehan Qayyum, Michael Neale, Jason Reed, Amir A. Toor

**Affiliations:** ^1^Bone Marrow Transplant, Virginia Commonwealth University Massey Cancer Center, Virginia Commonwealth University, Richmond, VA, United States; ^2^Virginia Commonwealth University Center for the Study of Biological Complexity, Virginia Commonwealth University, Richmond, VA, United States; ^3^Center for International Blood and Marrow Transplant Research, Minneapolis, MN, United States; ^4^Department of Microbiology and Immunology, Virginia Commonwealth University, Richmond, VA, United States; ^5^School of Pharmacy, Virginia Commonwealth University, Richmond, VA, United States; ^6^Mayo Clinic, Rochester Minnesota and King Faisal Research Hospital, Riyadh, Saudi Arabia; ^7^Department of Internal Medicine, Virginia Commonwealth University, Richmond, VA, United States; ^8^Department of Psychiatry & Statistical Genomics, Virginia Commonwealth University, Richmond, VA, United States; ^9^Department of Physics, Virginia Commonwealth University, Richmond, VA, United States

**Keywords:** stem cell transplantation, GVHD, antigen response, HLA, tensors, vectors and operators, matrices, T cell response

## Abstract

Alloreactivity compromising clinical outcomes in stem cell transplantation is observed despite HLA matching of donors and recipients. This has its origin in the variation between the exomes of the two, which provides the basis for minor histocompatibility antigens (mHA). The mHA presented on the HLA class I and II molecules and the ensuing T cell response to these antigens results in graft vs. host disease. In this paper, results of a whole exome sequencing study are presented, with resulting alloreactive polymorphic peptides and their HLA class I and HLA class II (DRB1) binding affinity quantified. Large libraries of potentially alloreactive recipient peptides binding both sets of molecules were identified, with HLA-DRB1 generally presenting a greater number of peptides. These results are used to develop a quantitative framework to understand the immunobiology of transplantation. A tensor-based approach is used to derive the equations needed to determine the alloreactive donor T cell response from the mHA-HLA binding affinity and protein expression data. This approach may be used in future studies to simulate the magnitude of expected donor T cell response and determine the risk for alloreactive complications in HLA matched or mismatched hematopoietic cell and solid organ transplantation.

## Introduction

Graft-vs.-host Disease (GVHD) represents a significant cause of morbidity and mortality in stem-cell transplant (SCT) recipients (1). GVHD in an HLA-matched allogeneic stem cell transplant is the archetype of an adaptive immune response with donor derived T cells responding to recipient antigens presented on shared HLA class I and class II antigens (2–4). HLA matching has been the bedrock principle of donor selection in SCT, and this is particularly so when the donor is not a close relative (5, 6). Improvements in the fidelity of HLA matching between unrelated transplant donors and recipients has yielded incremental benefits in patient outcomes, with improvements in survival resulting from both a reduction in GVHD risk, as well as reduction in graft loss and optimization of relapse risk. Nevertheless, GVHD remains a therapeutic challenge, and there is little that can be done to predict the outcomes of specific donor-recipient pairs.

This challenge may be surmounted by accounting for genomic variation between the donors and recipients which yields the peptides presented on HLA molecules, known as minor histocompatibility antigens (mHA) (7). While mHA have had a recognized pathophysiologic role in allogeneic SCT outcomes, especially in GVHD pathogenesis, it has not been possible to apply the notion to clinical practice because mHA characterization is a cumbersome process (8–11). Two developments in the past decade have potentially changed this situation. First, the emergence of next generation DNA sequencing techniques, such as single nucleotide polymorphism mapping (12, 13) and whole exome sequencing (WES) to identify the potential antigenic differences (14, 15). The second is the development of machine learning algorithms which allow determination of the binding affinity that different antigens may have for specific HLA molecules (16–18). These two techniques have been combined to develop algorithms that may be used to determine the complex array of recipient antigens that a given donor's T cells may encounter in a recipient (19, 20). Studies reporting exome-wide or other genomic disparities in donors and recipients, have demonstrated a large body of DNA sequence differences between transplant donors and recipients, independent of relatedness and HLA matching (13–15). These large genomic differences have been translated to peptides and HLA class I binding affinities for the resulting peptides determined (19). This too yields large libraries of antigens which may be analyzed by either simulating alloreactive donor T cell responses to these recipient antigens or by more conventional statistical methodology to determine predictive power for alloreactive T cell responses (21, 22). To date, these models have examined recipient peptide presentation on HLA class I and studied the resulting associations. Thus far, no obvious linear relationship has been identified between the magnitude of antigen burden and occurrence of clinical alloreactivity.

As noted above, HLA-matched SCT remains fraught with uncertainty as patients with HLA-matched donors continue to have disparate outcomes (23, 24). A quantitative model of transplant alloreactivity would allow a more complete understanding of the molecular immunology of SCT, potentially help to identify the most suitable donors for specific recipients, and allow personalized determination of the optimal level of immunosuppression. A central assumption in one such quantitative model, the dynamical system model of T cell responses, is that alloreactivity (such as GVHD) risk is a function of the cumulative mHA variation in the context of the HLA type of each donor-recipient pair (DRP) and may thus be regarded as an alloreactivity potential for that pair (14, 19, 25). Clinical outcomes partially depend on the cumulative donor T cell responses to the burden of polymorphic recipient peptides. Previous work applying this dynamical system model to HLA class I presented molecules demonstrates that there are large differences in the simulated T cell responses between different HLA matched DRP (21, 22). In this hypothesis developing paper, previously reported findings of WES of SCT DRP are extended with an analysis of the HLA class II presentation of polymorphic peptides. A comparison of the difference in magnitude of the *derived* peptide libraries presented on the HLA class I and HLA class II molecules in the DRP is presented. Next, a hypothetical quantitative model is developed which may allow the prediction of alloreactive T cell responses to similar large antigen arrays and their eventual application in clinical medicine. The mathematics introduced in the previously reported dynamical systems model of alloreactive T cell responses is generalized to include both HLA class I and HLA class II presented peptides. The model is expanded to account for different variables which may influence antigen-driven proliferation of T cells, including their own state of antigen-responsiveness and the cytokine milieu. This model may, in the future, permit successful simulation of alloreactive T cell responses between different donors and recipients in SCT.

## Methods

### Whole exome sequencing

**Figure 1:**
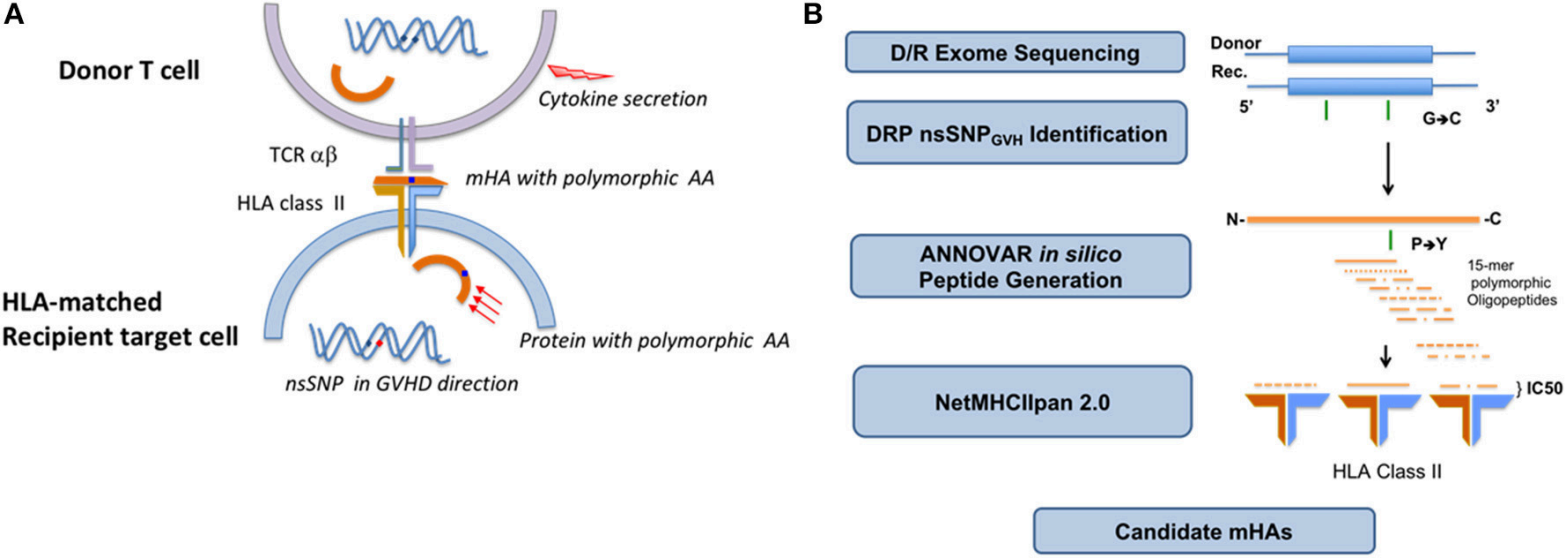
Non-synonymous single nucleotide polymorphisms present in the recipient and absent in the donor yield alloreactive peptides which may be presented to the donor T cells on HLA class I and II molecules. HLA class II presentation and CD4+ T cell recognition and response depicted **(A)**. Schematic depicting the analytic sequence from exome sequencing to HLA class II mHA prediction **(B)**.

After obtaining approval from the institutional review board (IRB) at the Virginia Commonwealth University (VCU), whole exome sequencing (WES) was performed on previously cryopreserved DNA samples from 77 HLA-matched DRP (Supplementary Table 1) as previously described (14, 21). Patients with recurrent or high-risk hematological malignancies undergoing allogeneic SCT at VCU were included in this retrospective study. DNA samples were de-identified by clinical research staff, and submitted for sequencing. The VCU IRB waived the need for informed consent on all adult participants as samples were all archived and previously de-identified, with only VCU BMT clinical research staff retaining access to any patient specific information involved in the retrospective analysis. The Sequencing team did not have access to the sample identity and clinical team did not have access to exome sequencing data. Nextera Rapid Capture Expanded Exome Kit was used to extract exomic regions from the deidentified DNA samples, which were then multiplexed and sequenced on an Illumina HiSeq 2500 to achieve an average coverage of ~90X per sample. 2X100 bp sequencing reads were then aligned to the human reference genome using BWA aligner. Duplicate read alignments were detected and removed using Picard tools. Single nucleotide polymorphisms (SNPs) in both the donor and recipients' exomes were determined using GATK HaplotypeCaller walker. GATK best practices were then implemented to filter and recalibrate the SNPs; and store them in variant call file (VCF) format. To identify SNPs unique to the recipient and absent in the donor the results from the GATK pipeline in VCF format were then parsed through the in-house TraCS (Transplant pair Comparison System) set of perl scripts. TraCS traverses through the genotypes of the called SNPs, systematically excluding identical SNPs or editing them to align with the graft-vs.-host (GVH) direction thereby generating a new VCF with SNPs for a particular DRP in the GVH direction (SNP present in the recipient, absent in the donor; R^+^/D^−^). All non-synonymous single-nucleotide polymorphisms (nsSNPs) present in the recipient and donor were identified and recorded in the.vcf format. Subsequent processing of the.vcf files was done using custom python scripts to remove synonymous mutations, eliminate duplicates, and record the coordinates of the SNPs. Non-synonymous SNPs that exist in the recipient but not in the donor were recorded and identified as a potential source of alloreactive antigens. Non-synonymous, single nucleotide polymorphisms (nsSNP) in each DRP would correspond to potential antigens due to the resulting amino acid substitution in oligopeptides which bind HLA in that DRP (Figure 1A).

### *In silico* determination of alloreactive peptide sequences

HLA class I bound 9-mer peptides were generated as previously described (19). Briefly, the SNPs in the VCF were annotated either as synonymous or non-synonymous using Annovar. For HLA class I binding affinity determination, the corresponding amino acid polymorphisms along with flanking regions of each protein were extracted using Annovar to build peptide libraries of 17-mers for each DRP, with the SNP encoded AA occupying the central position. This library was further expanded by sliding a 9-mer window over each 17-mer such that the polymorphic amino-acid position changes in each 9-mer. This resulted in the generation of 9 nona-meric peptides/SNP. The HLA class I binding affinity and IC50 values, which quantify the interactions between all these 9-mers for each DRP and all six HLA class I donor molecules (HLA-A, B, and C) was determined; NetHMCpan version 2.8 was run iteratively in parallel mode on a linux cluster using custom python scripts. Parsing the NetMHCPan output, unique peptide-HLA combinations present in the recipient but not in the donor, i.e., possessing a GVHD vector, were identified and organized in order of declining mHA-HLA affinity. To derive the peptide sequences bound to HLA class II molecules an average peptide length of 15 amino acids was used (26). Each of the nsSNPs could potentially be incorporated into the alloreactive peptide of 15 amino acids. The position of the nsSNP encoded polymorphic amino acid in the peptide could vary from the N-terminus to the C-terminus of the peptide. The possible library of peptides will thus be contained within a 29-mer oligopeptide (Figure 1B). Thus, there are 15 different HLA-II binding peptides that could potentially be generated from each nsSNP identified by WES. ANNOVAR was used to generate 29-mer peptides for each nsSNP respectively to study HLA class II presentation. In ANNOVAR, a sliding window method was used with the “seq_padding” option of the “annotate_variation” function to generate the 15 different 15-mers resulting from each nsSNP. Tissue expression of the proteins from which the peptides were derived was determined as previously described (22). Briefly, the Genotype-Tissue Expression (GTEx) portal V6 has publicly available expression level information (Reads/kilobase of transcripts/million mapped reads, RPKM values; http://www.gtexportal.org/home/) for a variety of human tissues over a large number of genes. Since the gene-ids for the proteins that generate the peptides in the DRP peptide library are known, the RPKM values from the GTEx portal for the specific gene across the whole array of tissues of interest were parsed in, namely, skin, lung, salivary gland, esophagus, small intestine, stomach, colon, and liver.

### Calculating HLA binding affinity of alloreactive peptides

Once the peptide library was created for each DRP, the HLA types for the recipient were tabulated from the medical records. HLA matching was performed using high resolution typing for the unrelated donor SCT recipients, and in related donor recipients intermediate resolution typing was performed for HLA class I, and high resolution typing for class II antigens. For class II HLA, HLA-DRB1 alleles for each patient were recorded. Each patient's HLA-DRB1 allele types (and HLA class I alleles, as previously described) along with peptide library were analyzed using NetMHCIIpan 2.0 to derive the binding affinity of each peptide-HLA complex. This was given as an IC50 (half-maximal inhibitory concentration) for each peptide, measured in nano-Molar. This measure of binding affinity provided the concentration of peptide required to displace 50% of a standard peptide from the HLA type to which it would have been bound.

### Data analysis

Peptides present in the recipient but absent in the donor, generated from the ANNOVAR sliding window with IC50 values for all the different patient HLA types were tabulated and duplicates were deleted. Any peptide with the same amino acid sequence but different SNP position along the peptide must have generated from a different area of the exome and was therefore retained in the enumeration. When compiling the peptides binding to different HLA alleles, the patients with homozygous allele for DRB1 had their peptide values doubled to simulate having double the normal number of allele-specific HLA bound peptides presented. Analysis of the number of strongly bound (SB; IC50 ≤ 50 nM) and bound peptides (BP; IC50 ≤ 500 nM) for each patient-HLA allele combination was done by listing the peptides in descending order of binding affinity, as measured by IC50 levels (Tables 1A,B). HLA class I and HLA class II bound peptides were compared numerically for this perspective paper.

Table 1Example of the strong binding peptides for HLA class II **(A)** and HLA class I **(B)** in a DRP.

**Table:** 

**Peptide**	**Seqid**	**Geneid**	**HLA-DRB1 03:01**	**HLA-DRB1 04:01**	**Colon**	**Esophagus**	**Liver**	**Lung**	**Salivary gland**	**Skin**	**Small intestine**	**Stomach**
**A**												
VLA**L**TYDSARLRWYF	s01086	GPR37	12.1	177.1	0.4	2.2	4.6	0.2	0.6	1.2	0.3	0.6
QHRLRLRA**Q**MRLRRL	s04022	AEBP1	12.7	515.4	62.0	51.0	8.0	113.5	35.0	53.0	51.1	31.7
AWLLLRSL**P**RRYIIA	s04661	SLC22A4	12.7	46.2	0.9	0.3	0.3	1.6	1.3	1.0	3.3	0.4
HRLRLRAQM**R**LRRLN	s04022	AEBP1	12.9	590.0	62.0	51.0	8.0	113.5	35.0	53.0	51.1	31.7
RLRLRAQMRL**R**RLNA	s04022	AEBP1	13.1	690.0	62.0	51.0	8.0	113.5	35.0	53.0	51.1	31.7
TAWLLLR**S**LPRRYII	s04661	SLC22A4	13.1	37.4	0.9	0.3	0.3	1.6	1.3	1.0	3.3	0.4
GNSSII**A**DRIALKLV	s07642	MTHFD1	13.1	129.7	8.7	10.6	44.2	8.8	7.2	9.7	9.2	8.6
HNRFRTLPPAL**A**ALR	s02269	RABGGTA	297.0	9.6	8.8	9.4	6.4	13.4	12.9	12.8	13.0	11.8
SHNRFRTLPP**A**LAAL	s02269	RABGGTA	376.3	9.9	8.8	9.4	6.4	13.4	12.9	12.8	13.0	11.8
NRFRTLPPALAA**L**RC	s02269	RABGGTA	367.9	10.7	8.8	9.4	6.4	13.4	12.9	12.8	13.0	11.8
LSHNRFRTL**P**PALAA	s02269	RABGGTA	653.9	12.5	8.8	9.4	6.4	13.4	12.9	12.8	13.0	11.8
PL**A**LQFLMTSPMRGA	s06833	TCN2	129.7	14.1	12.9	7.3	3.7	27.2	8.3	5.5	38.2	10.7
LAL**Q**FLMTSPMRGAE	s06833	TCN2	121.2	14.1	12.9	7.3	3.7	27.2	8.3	5.5	38.2	10.7
ISWFSSLLNNKH**F**LI	s03948	PLXND1	240.9	14.2	10.3	9.8	4.4	40.8	8.7	11.8	13.3	9.1
RFRTLPPALAALR**C**L	s02269	RABGGTA	542.7	14.6	8.8	9.4	6.4	13.4	12.9	12.8	13.0	11.8
LKEFYLT**R**NSPAEML	s00635	PARVG	429.4	14.7	1.9	1.2	1.6	12.0	1.9	0.6	7.9	1.7
ALQF**L**MTSPMRGAEL	s06833	TCN2	104.3	14.9	12.9	7.3	3.7	27.2	8.3	5.5	38.2	10.7

**Table:** 

**#peptide**	**Seqids**	**Geneids**	**HLA-A02:01**	**HLA-A30:02**	**HLA-B18:01**	**HLA-B55:01**	**HLA-C03:03**	**HLA-C05:01**	**Colon**	**Esophagus**	**Liver**	**Lung**	**Salivary gland**	**Skin**	**Small intestine**	**Stomach**
**B**																
YLFDVLPLL	s02238	OR8B4	1.7	3141.1	11193.9	19754.1	84.4	1250.2	0.0	0.0	0.0	0.0	0.0	0.0	0.0	0.0
ILMEHIHEL	s03554	RPL19	1.9	6234.5	13565.2	15481.0	30.2	1274.2	663.3	626.5	299.9	566.2	664.8	958.0	601.2	638.2
FMLFFIYAV	s01826	CACNA1S	2.2	6756.0	16867.4	11115.8	2958.2	12227.5	0.0	0.0	0.0	0.1	0.0	0.0	0.0	0.0
FMGDMLPSV	s02492,s06921	USP10	2.4	7164.3	27024.0	13985.6	989.7	3999.9	12.8	12.3	7.6	11.4	10.3	12.9	13.5	10.9
ALAPLAFFV	s04219	SLC16A13	2.5	2733.3	33198.9	22724.5	12376.0	4133.6	0.6	0.6	14.2	2.2	2.5	1.8	3.5	0.6
RQRSQFAFY	s05708	B4GALT5	19674.3	5.6	4627.8	10598.8	20895.8	27298.7	15.2	18.0	7.5	19.3	15.5	7.0	10.4	10.5
RSRRLFSHY	s02920	GNA15	30650.3	7.5	14677.8	10710.2	8741.3	14754.2	1.5	2.3	0.4	9.5	7.5	23.8	1.6	1.5
ASWTMSALY	s00607	OR7A5	21179.7	10.0	10536.7	14526.3	5981.8	6157.7	0.0	0.0	0.0	0.0	0.1	0.1	0.0	0.0
LSAFHYGLY	s01379,s02439	ABCA5	26537.2	10.4	7927.2	12597.3	4182.4	4691.6	4.5	4.9	8.6	3.9	7.1	10.8	12.7	6.5
RMTANHGSY	s03023,s03860	ARHGAP24	24064.5	12.6	10852.2	10622.3	5369.1	15075.7	2.2	2.3	0.9	4.3	2.2	2.9	3.1	4.4
YEYTGANVY	s00447	IBSP	33878.5	779.8	6.3	17946.0	5976.1	18402.2	0.0	0.0	0.0	0.0	0.0	0.0	0.0	0.0
IEYERFVPF	s03216,s05468	GRIA3	8426.0	7458.9	6.4	13617.5	2439.6	24416.2	0.3	0.1	0.4	0.1	0.1	0.3	0.2	0.3
TETEAIHVF	s00111	MUC16	31916.8	18682.9	7.6	34699.1	19947.8	23268.2	0.0	0.0	0.0	0.0	1.4	0.0	0.0	0.0
WEFCQAALF	s03238	STRC	20987.4	10333.5	8.1	28518.2	15505.0	24508.0	0.1	0.0	0.0	0.0	0.2	0.2	0.2	0.1
QEFPGSPAF	s01421,s04968	NUBP2	24752.6	10806.4	8.2	23170.9	4753.2	26150.7	11.1	11.3	12.2	10.3	9.7	12.7	9.9	11.7
HPYLPLVTA	s02088,s02121, s08500	RUNDC3A	17762.9	26351.5	12599.4	75.0	3424.7	28344.1	1.8	0.6	0.1	0.3	0.3	4.0	0.7	1.3
LPFFRSLPI	s00833,s01116, s01430,s01487, s02653,s03155, s03412,s03647, s04885,s05490, s06213,s06649, s07231,s08460, s08619	NR1I3	12522.5	16021.9	7734.8	89.1	244.5	19481.6	0.1	0.1	54.0	0.1	0.0	0.3	0.3	0.1
FPHYTPSVA	s05857	RNF43	30916.8	34577.0	19174.2	97.2	1693.0	24716.0	0.2	0.1	1.5	1.0	2.8	3.1	5.2	1.9
LPWLSHPSV	s00117	MUC16	9314.3	24690.0	10678.5	110.9	2683.1	22522.3	0.0	0.0	0.0	0.0	1.4	0.0	0.0	0.0
FPRSVNVTV	s01220	AZU1	19931.4	27730.1	14737.5	115.0	1100.3	19755.0	0.1	0.1	0.3	3.2	0.1	0.1	0.1	0.1
FSYPSSHPF	s06011	TAS2R31	1746.9	810.7	1838.5	3508.5	2.6	234.5	0.2	0.1	0.0	0.2	0.1	0.2	0.2	0.2
MAAPGSCAL	s04246	COQ5	2996.9	10729.4	12768.5	2263.3	2.9	850.6	12.2	9.9	18.1	10.3	10.1	11.6	12.5	10.8
FAHLSTYSL	s00176,s03760	CD200	723.1	11054.9	10504.8	1823.7	3.3	689.4	3.3	2.8	0.2	4.7	5.5	1.9	4.6	2.6
FSATAASSL	s05554	TNFSF12	4157.5	9673.6	23725.5	6769.4	3.5	233.5	51.6	54.7	9.1	42.1	18.1	16.2	24.5	21.9
YSSSGLSPM	s02382	OR13F1	2634.9	2264.7	10680.6	9430.4	3.6	299.1	0.0	0.0	0.0	0.0	0.0	0.0	0.0	0.0
YTDPYAQPL	s03106	KMT2D	92.3	5427.4	17458.2	18313.6	10.3	6.5	5.5	6.0	2.3	7.6	6.4	7.4	7.7	6.2
FSDEWVACL	s01960	GEMIN4	109.9	18368.6	25059.0	26436.0	77.7	8.8	4.6	5.5	2.4	5.2	5.6	11.4	5.4	6.9
LADEGTYEI	s00606	HEPACAM	339.0	22122.9	25359.9	11793.1	84.7	10.8	0.2	0.1	1.2	0.0	0.1	0.0	0.1	0.1
RTDPIQMPF	s05781	MPPED1	7092.1	400.9	21743.7	16422.4	251.3	11.3	0.0	0.0	4.6	0.0	0.4	0.1	0.0	0.2
ISDDTTQPI	s00689,s01247,s07200	GGT1	6127.1	14757.8	35688.4	20323.9	352.7	15.5	2.5	1.2	15.4	8.2	3.0	1.2	16.3	6.9

*Most peptides have relevant interaction with one HLA molecule but some bind multiple HLA molecules, more so with HLA class II molecules. The bold letters correspond to the polymorphic amino acid coded by the nsSNP*.

## Results and discussion

### HLA bound alloreactive peptides

Whole exome sequencing (WES) was performed on the cohort of 77 donor recipient pairs (DRP) of which 75 were evaluable for this analysis. SNPs were identified, following which alloreactive peptide binding affinities to HLA class I and HLA-DRB1 molecules were derived. There was marked variability in the number of peptides presented on the different HLA class I & II molecules between individuals in the cohort. An average of 1,085 ± 513 alloreactive peptides strongly bound (IC50 < 50 nM) all the HLA class I molecules/DRP, vs. 8,320 ± 11,158 peptides binding the two HLA class II molecules/DRP. When compared to an arbitrary sample with a standard deviation of 100 peptides, the variability observed in the test sample was highly significant (*P* ≤ 0.0001).

**Figure 2:**
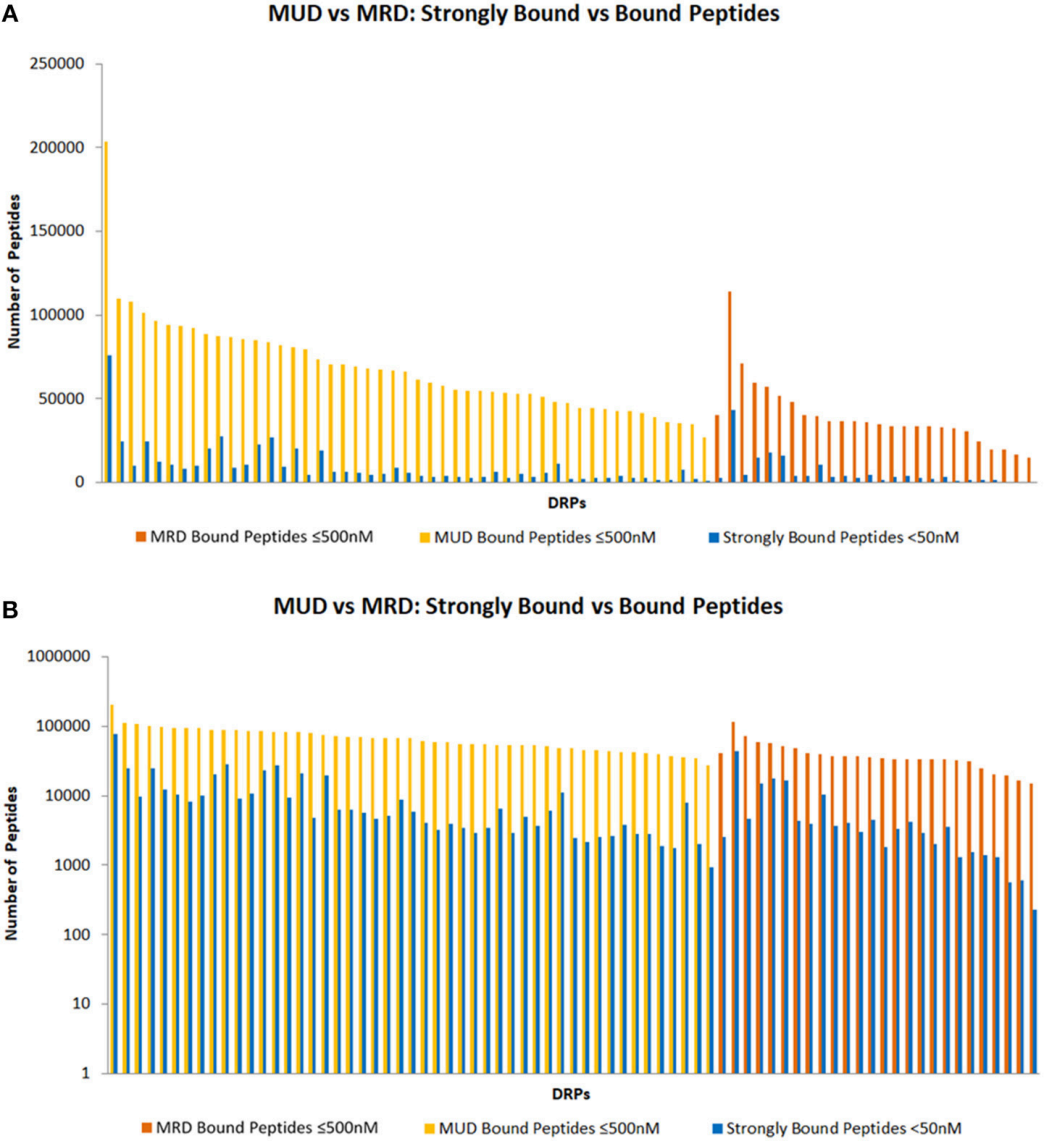
HLA class II bound peptides in HLA MRD and MUD DRP. Depicting SB and BP on standard **(A)** and logarithmic scales **(B)**.

In considering the HLA class II bound peptides, HLA matched unrelated donor (MUD) DRP exhibited a higher number of HLA-DRB1-bound peptide (BP); mean: 39,584 alloreactive peptides in HLA matched related donors (MRD) vs. 67,987 in MUD (*t*-test *P* < 0.001). When only the strongly bound (SB) peptides are analyzed, this trend while present, no longer remains statistically significant, mean SB 6,077 alloreactive peptides in MRD vs. 9,535 in MUD (*p* = 0.168) (Figures 2A,B). This is consistent with the larger burden of exome variation in MUD transplant recipients. Significantly more MUD DRP had BP > the median 52,983 peptides for the whole cohort (34/49 vs. 4/26, Fishers Exact test *p* < 0.0001), as well as SB >4,245 (30/49 vs. 8/26, *p* = 0.012), when compared to MRD DRP. There was marked variability in the HLA DRB1 allele binding affinity in the various peptides as well as the tissue expression of the proteins from which peptides were derived (Table 1A). This is likely an effect of the randomness observed in exome sequence variation, and the variation in HLA binding affinity of the resulting alloreactive peptides with different HLA molecules, and illustrates the potential for variability in alloreactive antigen presentation between different donors and recipients who undergo SCT.

### Computing donor T cell responses from recipient antigen arrays

These data illustrate the large potential that HLA class I and HLA class II molecules have for recipient peptide antigen presentation in the context of allogeneic SCT. Further complexity is added to the antigen driven donor T cell responses by variable tissue expression and different peptide cleavage potential (27, 28). Mass spectrometry studies have in general corroborated the presence of a large number of HLA class I and II bound peptides with several thousand unique peptides identified in studies of both malignant as well as non-malignant cell lines (29–31). These mass spectrometry studies also demonstrate significant variability in the distribution of presented peptides across different HLA class I and class II molecules, with antigen processing and post translational modification playing a role in antigen presentation, in addition to the antigen affinity of the HLA molecules (10, 32). All considered, the magnitude of this antigen burden across the patient population makes it difficult to predict which individual patient will have a poor outcome when utilizing simple statistically determined associations. However, while in and of themselves these parameters may not be definitive for GVHD prediction, given the uniformly large magnitude of mHA identified in the patient cohort examined, these measures if appropriately analyzed, may give insight into the quantitative principles governing the alloreactive T cell immune responses. To accomplish this, it is imperative to understand the quantitative principles at work in donor immune response and use these principles to develop methodology to simulate transplants with different donors *in silico*. The mHA prediction methodology presented previously and extended herein, augmented by analysis of peptide cleavage sites to more accurately determine the probability of the generation of specific HLA binding alloreactive peptides may allow prediction of alloreactivity potential for different DRP in the future (28). As a first step toward this goal, it was previously shown that donor CD8+ T cell growth simulations may identify patients at risk for moderate to severe GVHD, however these associations were relatively weak (22). While one possible explanation for this is the stochastic nature of alloreactive antigen presentation on HLA molecules (both alloreactive and non-alloreactive peptides may bind HLA), an important limitation in the special case of the model described (HLA class I antigen presentation) was its lack of information on HLA class II mHA presentation and consequent inability to simulate CD4+ helper T cell responses in the donor-recipient pairs involved. Normally, CD4+ T helper cells play an important role in the homing of cytotoxic T cells to infected tissues, and in the case of GVHD to the target tissues (33–35). In the transplant setting, T helper cells will recognize their target alloreactive antigens bound to HLA class II molecules; notably, these differ from the antigens recognized by CD8+ cytotoxic T cells and presented by HLA class I. The T helper cells initiate signaling by secretion of appropriate cytokines (IFN-?, IL-2, IL-12, IL-17 etc.) and set up the homing signal for the cytotoxic T cells to invade the target tissue, which cause tissue injury through direct cytolytic activity. In the present study we estimate the magnitude of alloreactive antigen burden encountered by donor cytotoxic T cells and helper T cells in HLA matched DRP. While this estimate is limited due to lack of protein cleavage site information, and absence of mass spectrometry verification, it may yet allow a more accurate calculation of the likelihood that a patient may develop T cell mediated tissue injury following SCT, then is possible with the current standard of conventional HLA matching.

### Comparing HLA class I and II bound alloreactive peptides

**Figure 3:**
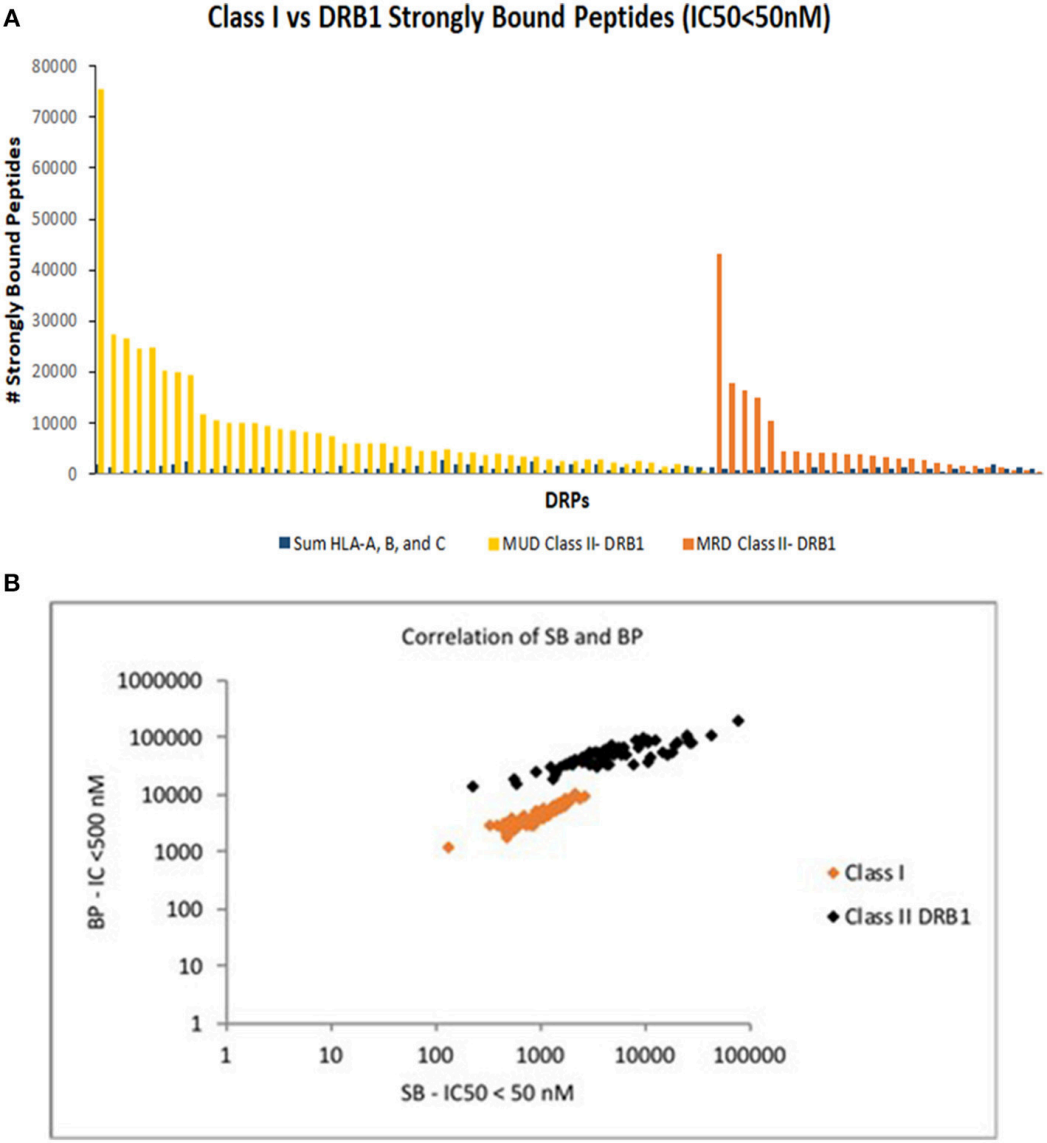
Comparison of HLA class I & class II bound peptides in HLA MRD and MUD DRP. Depicting SB peptides on standard scale in descending order and differentiated by donor type **(A)**. **(B)** Correlation of SB and BP for both HLA class I and class II molecules in MRD and MUD DRP.

How big a difference might the additional CD4+ T cell simulations make to the alloreactivity predictions of CD8+ T cell simulations performed earlier (22). To estimate this, the *in silico* derived HLA-DRB1 binding peptide libraries were compared to the numbers of BP and SB on all Class I HLA alleles for the same patients. On average, the number of alloreactive peptides bound to the two HLA-DRB1 alleles with an IC50 < 500 nM, was far greater than the number bound to the HLA class I loci (all 6 HLA-A, B, & C alleles). Significantly more peptides bound HLA DRB1 molecules compared to all the HLA class I molecules combined; BP for HLA DRB1 median 52,983 compared with BP for all HLA class I molecules 4,532, with a median ratio BP-HLA class I/BP-HLA DRB1 per DRP of 0.09 (range: 0.03–0.29; *t*-test *p* < 0.0001). The same association was observed with SB with a median ratio of 0.23 per DRP (range: 0.02–4.48; *p* = 0.0001) (Figure 3A). There was correlation between the number of BP and SB for both HLA class I and to a lesser extent in HLA class II molecules in the DRP studied; Pearson correlation coefficient, R 0.71, *p* < 0.0001 for HLA DRB1 & 0.94, *p* < 0.0001 for all HLA class I molecules together (Figure 3B). There was little overlap in the binding affinities of various alloreactive peptides to different HLA class I molecules (Table 1B). The difference observed in HLA class I and II antigen presentation using this computational methodology, may be related to the longer peptide length (15 amino acids) usually presented on the dimeric HLA class II molecules and used for calculations in this study. This increases the size of the peptide pool on offer (9-mer alloreactive peptides/SNP for HLA I vs. 15-mer for HLA II), and consequently the likelihood that alloreactive peptides will be presented. Therefore, validation of these findings utilizing mass spectrometry will be an important next step in this investigation.

Tissue expression of the proteins from which the peptides presented on HLA class I and class II were derived was also determined. In a subset of patients analyzed (*n* = 32), analogous to the variability in the number of peptides presented on HLA molecules. Indeed, there was a close correlation between the total number of SB peptides and the SB peptides expressed in each organ (*R* = 0.99 for both HLA class I and HLA class II molecules). There were differences observed in the expression levels of the proteins of origin for the alloreactive peptides (Tables 1A,B). However, when ordered by expression level as determined by RPKM values reported in the GTEX data base, the number of alloreactive peptides presented in different organs within an individual tended to be relatively similar (Supplementary Figure 1), hinting at the ubiquitous nature of proteins bearing polymorphisms. These overall *counts* of expressed peptides notwithstanding there is variation in the *level* of expression of different proteins, identifying this as another critical variable in simulating T cell responses.

### T cell clonal proliferation in response to mHA-HLA complexes: the logistic equation of growth

The relative differences in HLA class I and HLA class II peptide presentation observed here may contribute to the T cell subset repertoire diversity observed under normal circumstances (36). Previous work has shown there to be far greater diversity in the T cell repertoire of CD4+ T cells than in the CD8+ T cells in the post-transplant period in both allogeneic and autologous SCT (37), particularly when the memory T cell subsets are compared (38). In fact, CD4+ T cell diversity has been found to be about several times greater than CD8+ T cell diversity (39). This may be due to the ability of HLA class II molecules to present a larger number of peptide sequences compared to HLA class I molecules. The antigen-binding region of HLA Class II molecules consists of both an invariant α and a variable β domain, whereas that of HLA Class I molecules contains only α domains. This may allow HLA class II molecules to bind a wider range of peptide sequences (6, 40). This differential antigen presentation likely contributes to the quantitative difference observed between the two classes of T cells and may be understood using the dynamical systems approach. In this model, growth equations have been used to simulate the cytotoxic T cell growth in response to HLA class I presented antigen,

(1) $$\begin{array}{l} N_{t\;(T_{x})}=\frac{(P_{y}.{K_{\left(T_{x}\right)}}^{B_{y}Z_{x}})*N_{0(T_{x})}\;}{\left((P_{y}.{K_{\left(T_{x}\right)}}^{B_{y}Z_{x}})-N_{t-1(T_{x})}\right)(e^{-rtB_{y}})+1}... \end{array}$$

**Figure 4:**
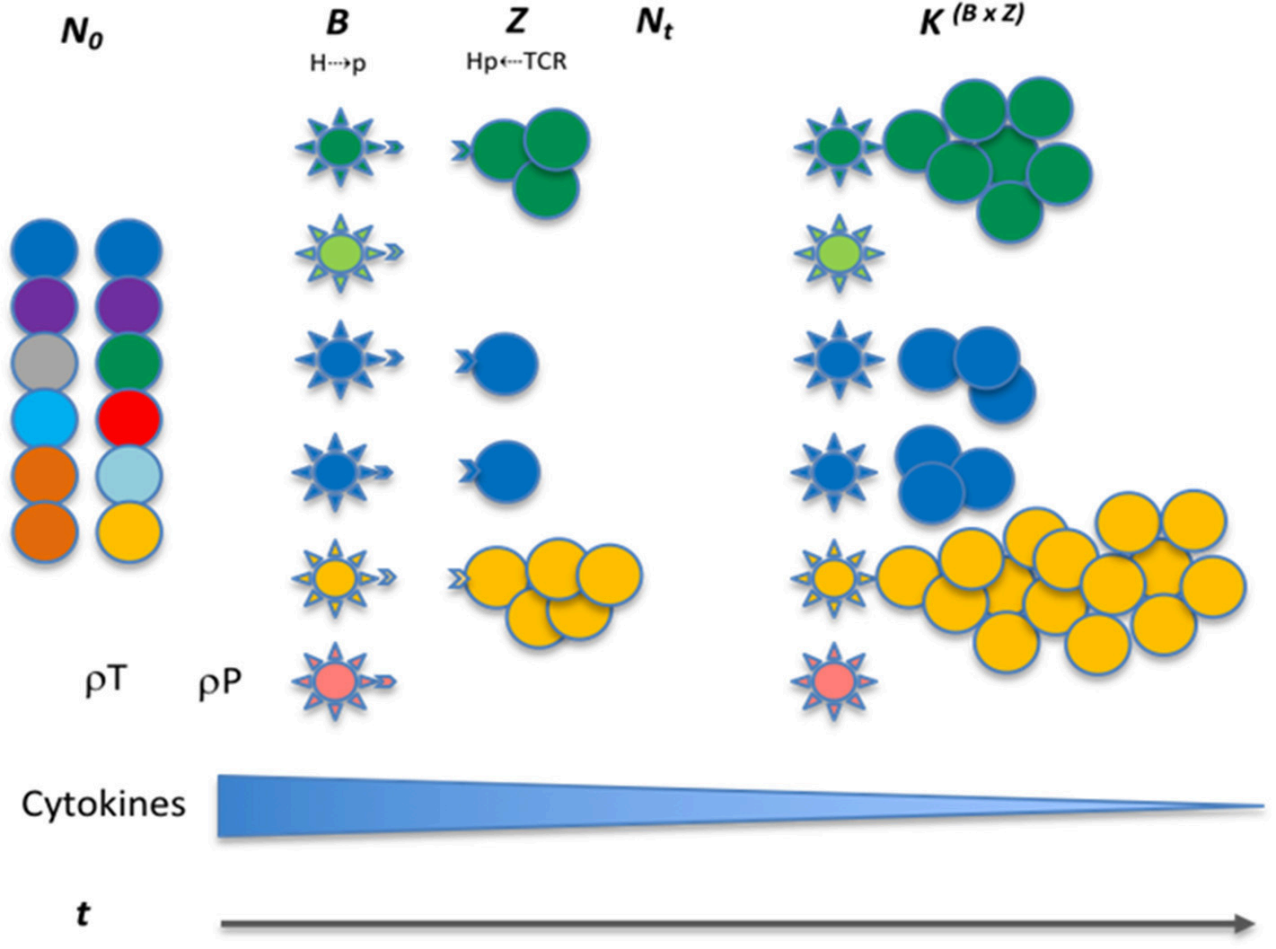
Interaction between donor T cells and recipient antigens presented on APC. Colored circles show different T cell clones with variable antigen affinity. The spiked spheres show antigen presenting cells with HLA molecules, colors indicate unique peptide antigens and correspond to T cell recognition. T cell growth is indicated over time in response to various influences, i.e. peptide affinity for HLA molecules (Vector *B*), and TCR affinity for HLA-peptide complex (vector *Z*), both leading to T cell growth. The infused allograft contains T cells with TCR of varying peptide antigen specificity (different colors), these may encounter peptides for which they have affinity (no color difference between APC and T cells), leading to growth. This variability makes antigen response a probability function of the likelihood of T cell presence and peptide antigen presentation (ρ*P &* ρ*T*). The expression level is depicted by more cells presenting the same antigen, making up for weak affinity driving proliferation. The cytokines made by APCs get taken up by the cytokine receptors on the T cells, leading to a diminishing effect as the T cell repertoire expands.

This iterating equation describes the logistic growth of a CD8+ T cell clone *T*_*x*_ in a polyclonal T cell graft infused into a recipient (Figure 4, Supplementary Table 2). *N*_0__(Tx)_ is the T cell count at the time of transplantation (assumed to be 1 for this equation), *N*_*t*__(Tx)_ is the T cell count after *t* iterations (time) following SCT. *N*_*t*−1__(Tx)_ represents the T cell count for the previous iteration and *K* is the proliferation constant that will determine the T cell count at the asymptote (steady state conditions after infinite iterations), *K*_(Tx)_, representing the maximum T cell count the *system* would support (*carrying capacity*); *r* is the growth rate. In the logistic equation, the steady state count for each T cell clone (*K*^*BZ*^) will be proportional to the *product* of the binding affinity of the target peptide mHA (peptide *y*) for the HLA molecule (*afmHA* = 1/IC50 in Koparde et al., in this paper, *B*_*y*_ for peptide *y*) and the affinity of T cell clone, *T*_*x*_'s T cell receptor for the mHA-HLA complex (*afTCR* = *1/IC50* in Koparde et al., now *Z*_*x*_ for T cell clone *Tx*) (22, 41). In its current form this equation assumes uniform expression levels of the T cell receptors. Presumably in activated T cells, TCR *may have a higher level*s of expression. Since this equations describes the expansion of T cell clones in response to the cognate antigens, it is likely that higher level of TCR expression may increase the probability of the T cell clones interacting with the APC that present the relevant antigens. In this model, the parameter *r*, determines the growth rate of the specific clone and reflects the effect of the co-stimulatory molecules and cytokines driving T cell proliferation. This iterating equation gives instantaneous T cell count (magnitude of the proliferative response) in response to antigens presented. The tissue expression of proteins from which peptide *y* is derived (*P*_*y*_) is a coefficient/multiplier for the steady state T cell population *K*^*BZ*^, and may be estimated by RNA sequencing techniques, and reported as Reads or Fragments Per Kilobase of transcript per Million mapped reads (RPKM or FPKM) (42). In real-world situations the term *P*_*y*_ will have a time modifier, *e*^*t*^, associated with it, as protein expression and antigen amount declines over time because of tissue injury. This time relationship will be ignored for simplicity at this time. It is important to recognize that in HLA class I-presented antigen-driven T cell expansion, this term is utilized in its entirety given that HLA class I molecules are loaded using peptides derived from proteins present in the cytosol. This however is not the case for HLA class II molecules, which generally present antigens endocytosed from the extracellular environment (43). This means that when calculating helper T cell growth, the term *P* will be modified to *P.c*, with a constant, *c*, reflecting the attenuation of antigen concentration given its “scavenged” nature as opposed to direct cytosolic presence, in other words, *0* < *c* < *1* (for CD8+ T cells, *c* = *1*). Thus, the equation for determining helper T cell growth will take the general form,

(2) $$\begin{array}{l} N_{t\;(T_{x})}=\frac{(P_{y}c.{K_{\left(T_{x}\right)}}^{B_{y}Z_{x}})*N_{0(T_{x})}\;}{\left((P_{y}c.{K_{\left(T_{x}\right)}}^{B_{y}Z_{x}})-N_{t-1(T_{x})}\right)(e^{-rtB_{y}})+1}\ldots \end{array}$$

Adjusting the variable *P* means that the absolute magnitude of the steady state T cell population for each of the dominant (high-ranked) helper T cell clones will be smaller than that for each of the dominant cytotoxic T cell clones, nevertheless because of the greater number of possible antigens presented by HLA class II molecules there may be a greater number of CD4+ T cell clones, and thus greater clonal diversity of helper T cells when compared to cytotoxic T cells. This also means that in a Power law clonal frequency distribution analysis (44, 45), the contribution of the highest-ranking (most numerous) T cell clones to the entire repertoire will be higher with cytotoxic T cells (38). Conversely, in the T helper cell population there will be a larger number of high-ranking clones which contribute a larger component of the overall repertoire. Given the greater number of antigens there may be greater competition between the clones, which in a model accounting for competition between clones will lead to slower growth of helper T cell clones, a relatively frequent clinical observation (46). Also, given the restriction of HLA class II molecules to antigen presenting cells the absolute magnitude of steady-state helper T cell clonal populations will be smaller; however, since HLA class I molecules are expressed on all nucleated cells, cytotoxic T cells get a proliferative signal from many different cell types, therefore steady state T cell clonal counts can be further augmented. From an evolutionary and T cell response sensitivity and specificity standpoint, it is logical that the cytotoxic T cell-recruiting signal provided by CD4+ T helper cells should be more sensitive, triggered by a greater variety of antigens, but when it comes to actual tissue destruction by CD8+ cytotoxic T cells, a more fine-tuned HLA class I bound, shorter peptide with greater specificity required for presentation, provides the necessary stimulus. This would come from the prevention of non-specific binding of peptide antigens to the more “discriminating” HLA class I molecules.

### Overlap between HLA class I and II peptide pools

To determine if there is overlap between HLA class I and II presented peptides, two DRP (one each MRD and MUD), was analyzed to determine the likelihood of peptide presentation from the same proteins on both HLA class I and II molecules. This would result in activation of both CD4+ and CD8+ T cells in the tissues expressing that protein, and greater potential for tissue injury. A comparison of strongly bound peptides (IC50 ≤ 50 nM) demonstrates that these DRP had 143 and 343 genes respectively, that yielded peptides binding both HLA class I and HLA class II. Different degrees of sequence of homology between these 9-mer and 15-mer peptides was observed (Table 2, Supplementary Figure 2). This overlap suggests that if the degree of exome sequence variation in a DRP is sufficiently large, it is plausible that most tissues will potentially present mHA to both helper and cytotoxic T cells. It is also important to note that such protein expression overlap in different tissues in addition to facilitating T helper and cytotoxic T cell interactions, may impact regulatory T cell function such that it serves a protective function against cytotoxic T cells which may recognize alloreactive antigens in a particular tissue. An example of such effect will be the clinical benefit of interleukin-2 observed in chronic GVHD (47).

**Table 2:** MRD DRP 26, polymorphic HLA-bound (both class I & II) peptides derived from the same gene, with binding affinity values in the IC50, 0–50 nM range.

**Geneids**	**Class I 9-mer**	**HLA-A02:01**	**HLA-A30:02**	**HLA-B18:01**	**HLA-B55:01**	**HLA-C03:03**	**HLA-C05:01**	**Class II 15-mer**	**HLA DRB1 0301**	**HLA DRB1 0401**
AGXT2	**FAVEVFRSA**	1516.1	19896.3	12500.5	1455.5	40.2	10055.5	**FAVEVFRSA**LTQHME	460.1	28.9
AK9	**FLMNPRPYL**	4.3	4760.2	20565.6	7448.6	25.3	1336.9	ALKP**FLMNPRPYL**LP	29.3	193.0
ASPM	**ASIVIQSTY**	31629.4	44.2	13973.2	18924.6	4613.3	10787.6	HK**ASIVIQSTY**RMYR	25.7	60.0
AVPR1A	**FGMFPSAYM**	3674.0	3498.8	12767.8	7866.3	11.4	2197.5	HLQV**FGMFPSAYM**LV	2331.0	43.9
CATSPERD	**YSLTAQSAM**	10576.8	4156.0	12288.7	8996.0	8.7	1407.0	SYS**YSLTAQSAM**CTS	2896.8	41.7
MC1R	ISIF**YALRY**	25706.3	31.9	8957.9	19531.2	5801.1	9626.4	**YALRY**HSIVTLPRAR	135.3	18.1
NOP56	**KTRGNTPK**Y	35107.0	15.5	27598.1	14424.7	9963.6	22714.2	KALFRAL**KTRGNTPK**	994.0	41.2
OR1I1	QL**LDVYHVL**	11.6	8139.2	14953.2	30075.2	755.1	11126.0	**LDVYHVL**GSLLAARD	846.9	47.0
OR4C3	**TAPAFSVT**L	5503.3	20654.6	22712.2	25030.0	43.7	1962.4	LLVFIGN**TAPAFSVT**	462.0	40.8
OR2T8	L**LIHWDHRL**	20.0	10956.6	27156.7	27657.6	1524.5	8839.8	**LIHWDHRL**HTPMYFL	36.4	842.3
DENND3	**FVMAPTSFL**	10.3	3652.5	17042.6	2484.8	4.5	389.9	MLD**FVMAPTSFL**MGC	238.6	17.4
FAM186A	**HMDTVQLGY**	11356.2	49.5	6536.0	21226.9	9349.7	1234.1	EIL**HMDTVQLGY**LFR	46.0	109.9
KRT19	**VSSSSSGAY**	35731.6	24.1	14800.9	14059.5	2267.3	3801.6	ARF**VSSSSSGAY**GGG	3454.2	27.7
OTOF	**MSNNKRVAY**	30323.2	41.0	4779.4	6417.7	1125.4	7254.6	FIWM**MSNNKRVAY**AR	71.8	37.8
TUBA3E	**LMYAKSAFV**	5.0	2233.4	26897.4	10045.0	1579.9	6248.2	KFD**LMYAKSAFV**HWY	287.6	40.3
EXOC4	FLNMVCEKL	33.8	17747.3	28320.8	34391.9	2684.4	4867.5	ELEYIHALTLLHRSQ	42.4	19.3
MAGEL2	MVKVIHREY	30350.7	45.0	4482.6	8535.1	6459.2	30155.9	APAVIRQAPPVIRQA	28.1	116.4
MUC16	TETEAIHVF	31916.8	18682.9	7.6	34699.1	19947.8	23268.2	TSQGTFTLDSSSTAS	408.1	45.4
TMPRSS9	FLSTQVFHV	2.7	12190.6	29318.2	19445.1	3194.4	2402.1	ELRGIRWTSSFRRET	15.0	244.6
ZNF568	FSYDTQLSL	353.6	7765.1	17478.8	5206.5	3.8	150.4	GKAFSQSSSLTVHLR	450.8	33.1

*Varying degrees of sequence identity is seen between the HLA bound peptides; 5 peptides per degree of sequence homology are presented and homologous sequence is given in bold. Rows 1–5, 9-mer HLA class I-bound peptide nested within 15-mer HLA class II-bound peptide; rows 6–10, sequence overlap between 9- and 15-mer peptides; rows 11–15, variable sequence homology (at least 1 nested, shown, and at least 1 matched only by gene nearby with no sequence identity, not shown); rows 16–20, different HLA bound peptides from the same gene with no sequence identity (Supplementary Figure 1)*.

### Quantifying mHA-HLA-TCR interactions: on matrices, vectors, and tensors

Following the above general discussion about T cell behavior, it is necessary to develop a model that will account for the potentially large arrays of antigens being presented in allogeneic SCT as seen in the data sets reported in this paper. As noted earlier, immunotherapy and SCT are fraught with the risk of treatment failure either in the form of relapsed malignancy or immune mediated normal tissue injury (GVHD or graft rejection). Various outcome prediction algorithms and models have been developed using increasingly sophisticated characteristics studied statistically (48, 49). These may allow improvement in clinical outcomes prediction, but often do not provide mechanistic insight into the reason for the observed clinical outcomes. Further, while principles of immune therapy and the mechanisms of T cell action are well known from work on mouse models and *in vitro* (50, 51), when the antigenic complexity encountered *in vivo* in human SCT recipients is considered, the existing models do not reliably predict individual clinical outcomes. This is also true of the T cell repertoire that emerges following SCT.

Nevertheless, mathematical methods are available that have long been used in physics to understand natural phenomenon and may be extrapolated to biological systems such as immune response modeling. For example, the concept of vectors and operators has been used to simulate aggregate T cell clonal responses to antigen arrays (21, 22). However, this method is limited in that it requires identification of unique mHA-HLA and it's cognate TCR for application. To overcome this limitation, a related mathematical method, tensor analysis, may be used to simulate the entire T cell clonal response to the vast library of tissue specific antigens presented by the entire spectrum of HLA molecules in an individual. In physics, tensors describe interaction between vector quantities and their components, so they enable determination of variation in vector magnitude and direction and subsequent mapping to a different “state.” In other words, tensors help describe vector transformation when multiple forces are acting upon an object, which itself may be a vector (52–54). It is important to recognize that these methods have been developed for use in “linear” physical systems, however biological systems are seldom linear. They follow non-linear dynamics such as Power laws and exponential growth patterns, which require adaptation of this methodology to account for such behavior and the inherent complexity in biologic systems because of the multiplicity of variables encountered. It is because of it's adaptability that tensor methodology may lend itself to the study of the alloreactive immune response problem. In the example at hand, the donor T cell array infused into the recipient may be considered as a vector, which is modified by the interaction between the T cell receptors (TCR) on the donor T cell clones and the recipient mHA-HLA complexes and is transformed to a new state following SCT. The interacting TCR and mHA-HLA complex in this example may be considered as a tensor, modifying the T cell clonal vector. Tensors remain invariant in different frames of reference and in this application of the concept, the mHA-HLA-TCR interactions, determined by the protein sequences remain constant, regardless of tissues and individuals where the interactions may be occurring. In other words, the unique peptide sequences' affinity to specific HLA molecules and TCR will remain the same across individuals and tissues. In essence, such an alloreactivity tensor comprised of recipient mHA and HLA, in the presence of donor T cell repertoire influences the relative growth of alloreactive T cell clones vs. the non-alloreactive clones. Accordingly, clinical GVHD may or may not manifest.

**Figure 5:**
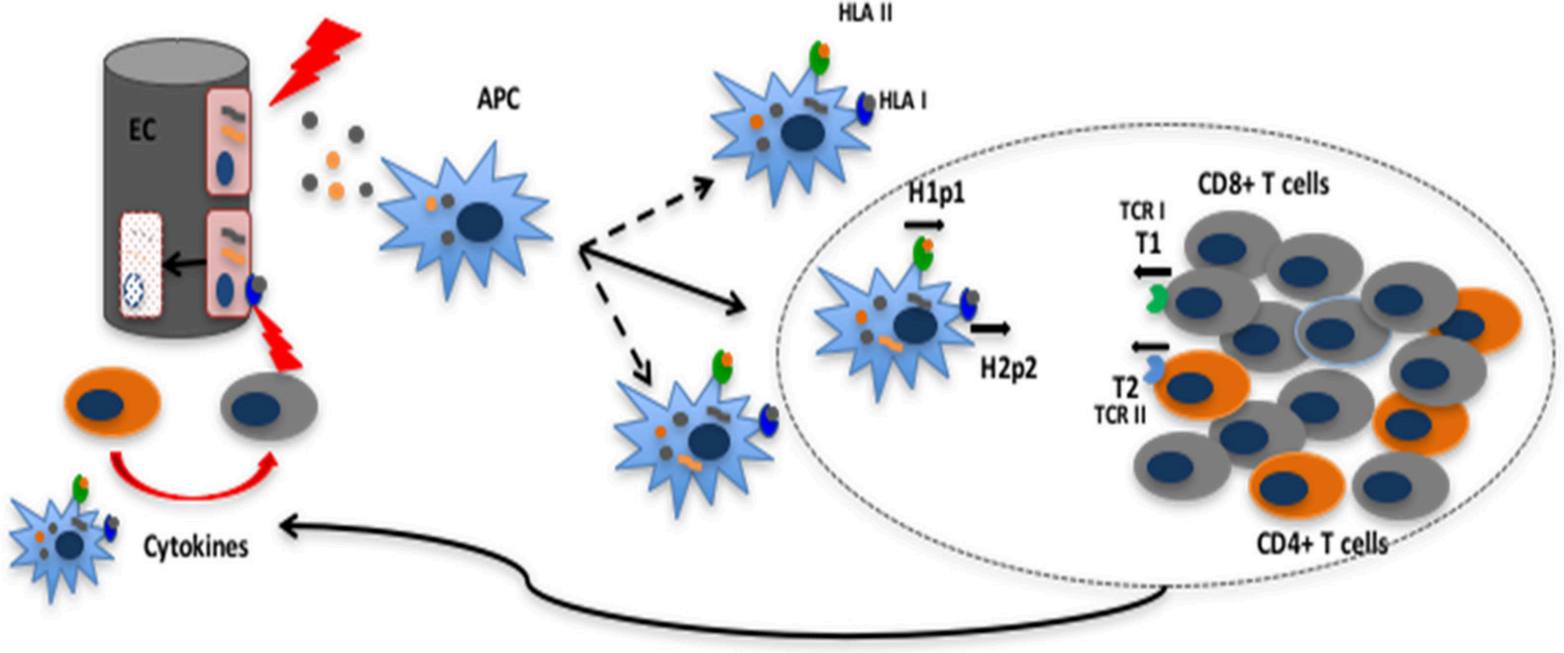
Tissue injury releases polymorphic recipient mHA from epithelial cells (EC); these and endogenous antigens are presented by APC; the APC proliferate and migrate to the lymph node triggering a CD4+ and CD8+ T cell clonal expansion according to the logistic equation of growth. These T cell clones then enter the circulation and migrate to the tissues to initiate tissue injury. Short black arrows in the oval (lymph node) below H1P1 and TCR1 indicate affinity vectors B and Z respectively.

To understand this notion, consider a basic adaptive immune response to a recipient alloreactive peptide following SCT (or any other antigenic peptide); the first interaction is between the alloreactive recipient peptide and the HLA molecule resulting in the binding and presentation of the peptide on the HLA molecules (Figure 5). Consider two HLA molecules *H*_1_ and *H*_2_, and two peptides *p*_1_ and *p*_2_, each recognized by only one of these two HLA molecules; a matrix may be constructed showing the peptides bound to the relevant HLA molecules (55). The possible interactions between the peptides *p*_1_ and *p*_2_ in a system of two HLA molecules *H*_1_ and *H*_2_, may be depicted in matrix form as follows.

(3) $$\begin{array}{l} \left(\begin{array}{c} H_{1}p_{1}\;H_{1}p_{2}\\ H_{2}p_{1}\;H_{2}p_{2} \end{array}\right)=\;\left(\begin{array}{c} 1\;0\\ 0\;1 \end{array}\right)\;\ldots \end{array}$$

The 0 and 1 represent conditionality of interaction between the peptides and HLA. The matrix on the left-hand side of Equation (3) represents vector quantities, *H*_1_*p*_1_*, H*_1_*p*_2_*, H*_2_*p*_1_, *or H*_2_*p*_2_, which have a magnitude (binding affinity, expressed in 1/IC50, nM^−1^) and a “direction” given by the specificity, i.e., unique affinity of the peptide for the HLA molecule. Given affinity of H_1_ for p_1_ and H_2_ for p_2_, this interaction yields an identity matrix. The interaction between the peptides and HLA molecules constitute a matrix where peptide recognition and binding by an HLA molecule is represented by 1, and the converse situation by 0. Thus, the numbers 1 & 0 represent the selectivity of peptides with a certain sequence (and commensurate length) for specific HLA and *vice versa*. These two alloreactive HLA-peptide complexes may then be presented to donor T cell clones by the antigen presenting cells, (Figure 5) and specific donor T cell receptors may recognize these unique HLA-peptide combinations and bind. In this example, *TCR*_1_ only recognizes *H*_1_*p*_1_ and, *TCR*_2_ only recognizes *H*_2_*p*_2_. The resulting matrices are given below

(4) $$\begin{array}{l} \left(\begin{array}{c} H_{1}p_{1}\;H_{1}p_{2}\\ H_{2}p_{1}\;H_{2}p_{2} \end{array}\right).\;\left(\begin{array}{cc} TCR_{1} & 0\\ 0 & TCR_{2} \end{array}\right)\\ \;=\;\left(\begin{array}{cc} H_{1}p_{1}.TCR_{1}+H_{1}p_{2}.0 & H_{1}p_{1}.0+H_{1}p_{2}.TCR_{2}\\ H_{2}p_{1}.TCR_{1}+H_{2}p_{2}.0 & H_{2}p_{1}.0+H_{2}p_{2}.TCR_{2} \end{array}\right)\\ \;=\;\left(\begin{array}{c} 1+0\;0+0\\ 0+0\;0+1 \end{array}\right)=\;\left(\begin{array}{c} 1\;0\\ 0\;1 \end{array}\right)=\left(\begin{array}{cc} HpT_{1,1} & 0\\ 0 & HpT_{2,2}\; \end{array}\right)\ldots \end{array}$$

The right-hand side of Equation (4) is a tensor with two vector quantities, the affinity of HLA molecule for the peptide and the affinity of the TCR for the peptide-HLA complex, which may be summarized as follows

(5) $$\begin{array}{l} \left(\begin{array}{c} HpT_{1,1}\;0\\ 0\;HpT_{2,2} \end{array}\right)=\;\left(\begin{array}{c} B_{1}\;Z_{1}\;0\\ 0\;B_{2}\;Z_{2} \end{array}\right)=\;\left(\begin{array}{c} 1\;0\\ 0\;1 \end{array}\right)\;\ldots \end{array}$$

**Figure 6:**
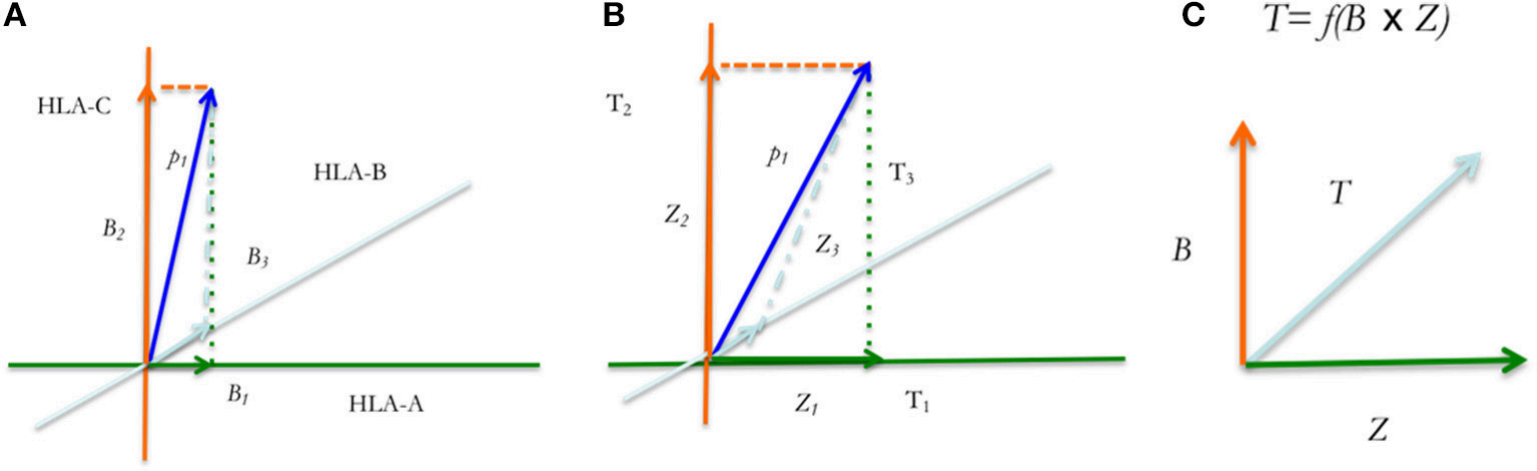
Tensor diagram for the HpT tensor. Peptide *p*_1_ binds different HLA molecules, **(A–C)** with affinities, B_1_, B_2_ and B_3_ **(A)**, while each TCR (TCR_1−3_) binds these HLA-peptide complexes (with peptide, *p*_1_), with affinities Z_1_, Z_2_, and Z_3_ **(B)**. The vectors *p1* indicate the average effect of multiple binding affinity vectors. These affinities will remain unaffected in different tissues and individuals. **(C)** T cell clonal growth in response to the polymorphic peptides is proportional to their product.

The matrix depicted in Equation (5), is a tensor of the second rank with two vector quantities, i.e., the affinities *B* and *Z* (specific binding between HLA & peptide (*B*) and between HLA-peptide & TCR (*Z*)), which are depicted by *HpT*_1,1_ and *HpT*_2,2_. *HpT* in this case symbolizes the HLA molecules, peptides and TCR interacting with each other, and the subscripts *1* and *2* are called indices in tensor terminology, identifying interactions between specific molecules (e.g., *p*_1_ and *p*_2_). The identity matrix reflects the affinity of specific TCR for specific mHA-HLA combinations. It is to be noted that, the same peptides given above may bind other HLA molecules with a different affinity and there may be TCR which bind these alternative antigen complexes with different affinities, constituting different vectors (56) (Figures 6A,B). Along the same lines, a given peptide or TCR may interact with different partners yielding different *vector components*. For example, in the above matrices, *TCR*_1_ may interact with both *H*_1_*p*_1_ and *H*_1_*p*_2_, the magnitude of the former will be 1 and the latter, 0. However, given the continuous nature of the IC50s observed for different peptides with different HLA molecules in the analysis presented in this paper it is unlikely that the vector magnitudes are going to be binary in nature. The well-known phenomenon of immune cross reactivity is an example of the vector components which are not binary (57). It is also important to note that the forces (vectors) represented by *B* (*H*_1_*p*_1_) and *Z* (*TCR*_1_) may be considered orthogonal (perpendicular) because their direction is imparted by the unique recognition of peptide sequence by HLA, and that of peptide-HLA complex by TCR respectively. Thus, the growth of the T cell clone resulting from this interaction may be considered a “cross” product of these two forces (*Sin 90*° = *1*, for orthogonal vectors) (Figure 6C).

### T cell vector transformation: enter operators

In the SCT context the alloreactivity tensor, *HpT*, determines the magnitude (and direction) of T cell clonal growth vector in response to antigens. T cell clones with receptors *TCR*_1_ and *TCR*_2_ respectively will grow in response to the *HpT* Tensor. It is to be noted that the HLA-peptide driven T cell clonal growth vector is distinct from the TCR affinity vector for HLA-peptide complex, even if one considers that mHA-HLA affinity vector drives T cell clonal growth of the relevant TCR bearing clone. This relationship is analogous to applied force, resulting in motion at a certain velocity and consequent mass displacement which are distinct vector quantities pointing in the same direction (with time being the scalar distinguishing between them; T cell clonal growth is also a time-dependent function). In the above example, the T cell clonal growth vectors, comprising the two T cell clones bearing the T cell receptors *TCR*_1_ and *TCR*_2_, are termed *T*_1_ and *T*_2_ respectively. These constitute a vector matrix, which is transformed over time *t* by the *HpT* tensor to the vectors *T*_1_' and *T*_2_'.

(6) $$\begin{array}{l} \left(\begin{array}{c} T_{1}^{\prime }\\ T_{2}^{\prime } \end{array}\right)\;=\frac{d}{dt}L\left(\begin{array}{c} B_{1}\;Z_{1}\;0\\ 0\;B_{2}\;Z_{2} \end{array}\right)*\left(\begin{array}{c} T_{1}\\ T_{2} \end{array}\right)\ldots \end{array}$$

In Equation (6), the vector $\left(\begin{array}{c} T_{1}\\ T_{2} \end{array}\right)$ *is* transformed by the *HpT* tensor and the logistic operator, $\frac{d}{dt}L$ previously defined as the logistic equation for T cell growth, which incorporates the term *B*_*y*_*Z*_*x*_ included in the *HpT* tensor,

(2) $$\begin{array}{l} N_{t\;(T_{x})}=\frac{(P_{y}.c.{K_{\left(T_{x}\right)}}^{B_{y}Z_{x}})*N_{0(T_{x})}\;}{\left((P_{y}.c.{K_{\left(T_{x}\right)}}^{B_{y}Z_{x}})-N_{t-1(T_{x})}\right)(e^{-rtB_{y}})+1}\ldots \end{array}$$

### T cell growth: the effect of co-stimulation, checkpoints, and cytokines

In Equation (2) the term *r* quantifying growth rate is an aggregate measure of different influences on T cells and may be considered a scalar multiple of a tensor quantity. This term represents the cumulative growth effect of the costimulatory and inhibitory molecules present on the T cells and the cytokines present in the environment. In the dynamical system model of T cell growth, the T cell steady state numbers are determined by TCR-mHA-HLA affinity (*BZ*), also called “Signal 1.” A second critical influence on T cell growth is provided by “Signal 2” mediated by the costimulatory molecule CD28 and inhibitory molecule CTLA4 (*S2*) may be mathematically represented by, CD28 = 1, CTLA4 = 0. Additionally, the checkpoint mechanism (*CP)* comprising the PD1 receptors, if engaged may be represented by a variable valued at 0 because no T cell growth will occur, and when absent, valued at 1. Finally, “Signal 3,” (*S3*) represents the effect of cytokines on T cell growth (Supplementary Figure 3) (58–60). Considering that all these variables contribute to T cell growth, the term *r* is therefore a composite of the following factors,

(7) $$\begin{array}{l} r=CP\left(S2*S3\right)\ldots \end{array}$$

Solving this equation for lack of PD1 engagement (1) and the presence of CD28 expression (1) yields,

$$\begin{array}{l} r=1\left(1*S3\right)\\ r=S3 \end{array}$$

Solving the equation for CTLA4 expression or PD1 engagement gives *r* a value of 0, which yields *e*^0^ = 1 in Equations (1, 2), consistent with suppression of T cell growth. In other words, the presence of PD1 engagement by PDL-1 or the engagement of CTLA-4 instead of CD28, by CD80 on APC, changes *r* to zero, eliminating the effect of time *t*, which changes the value of *e* to 1 (in Equation 2), leading to growth arrest of the T cell clone.

As for *S3*, the cytokine mediated signal may also be considered a second order tensor quantity, consisting of a matrix with cytokines and cytokine receptor vectors, because the cytokines and their receptors, have different magnitudes and varying receptor specific effects (directionality) on T cell growth and differentiation. Ignoring the di- or trimerization of cytokine-receptor protein subunits, a simplified version of the cytokine tensor may be constructed as follows,

(8) $$\begin{array}{l} \left(\begin{array}{c} IL12\;0\\ 0\;IL10 \end{array}\right).(\begin{array}{c} IL12R\;0\\ 0\;-IL10R \end{array}\;)\;\\ =\;\left(\begin{array}{cc} IL12.IL12R & 0\\ 0 & -IL10.IL10R \end{array}\right)\ldots .. \end{array}$$

This is the cytokine tensor, *Ck*, with the example showing the interaction between IL-12 and IL-10 and their respective receptors. It should be noted that cytokines may bind related receptors with different affinities, providing different vector components. The negative sign means a growth suppressive effect, the net effect of cytokines can either be negative or positive and as a multiple of the CD28-PD1 expression term, the *Ck* can alter the magnitude and direction of effect of the exponent in Equation (2) (by changing the symbol of *r* from – to +). Equation (7) therefore is modified to

(9) $$\begin{array}{l} r=\;CP\left(S2*\;Ck\right)\ldots . \end{array}$$

Further complicating these estimations from a physical standpoint at a cellular level in Equation (8), cytokine exposure will be variable since these effects are “local” to the tissue or lymph nodes. Cytokines likely depend on diffusion via capillary action in the extracellular matrix to create a “field” in which the T cells experience the cytokine effects. These effects on growth are of an exponential nature because of *r* being an exponent in Equations (1, 2) (61). The receptor expression levels also vary on different cells and confer a direction by means of influencing differentiation and functional specificity to the T cell clones with unique TCR.

### Evolution of the T cell repertoire: putting it all together

The above discussion illustrates the complexity inherent in the multiple factors influencing the T cell responses to antigens presented by HLA molecules. Nevertheless, it makes it clear that despite the complexity, it is possible to describe the immune interactions in mathematical terms, and therefore it is also possible to simulate them, especially when antigen presentation data are available. To do so one may take the example of a random collection of tissue associated peptides. First, consider an alloreactive peptide of any size varying between 7 and 18 amino acids. This peptide will have a choice of binding to HLA class I and II molecules (there are six of each). Therefore, depending on its size and mode of acquisition (extracellular or cytosolic) it will bind to the relevant HLA molecules with a unique binding affinity. It is to be noted that depending on the number of binding HLA molecules and the concentration of competing peptides, there will be a probability function associated with each of these interactions. As demonstrated above in Equations (4, 5), the mHA (polymorphic peptide) binding affinity to available HLA molecules, may be considered to represent the components of the immune response vector to this antigen (or degrees of freedom for the peptide). For most peptides, only one component (one HLA-mHA complex) with the strong interaction will be relevant, and others with weak interactions may be ignored. With the peptide bound to one of the HLA molecules (or more depending on binding affinity with other HLA molecules), it is presented on the APC. If a T cell clone with a TCR which has affinity for the HLA-peptide complex is present (a second probability term), then depending on the CD28/CTLA-4 and PD1 expression levels in the T cell clone, it will grow in the cytokine “field” present in the tissue.

Thus, consider peptides (*p*_1_*, p*_2._*. p*_*n*_) with high affinities *B*_1_*, B*_2_… *B*_*n*_ for HLA molecules *H*_1_*, H*_2_… *H*_*n*_ respectively, but with a very low-level affinity for the non-corresponding HLA molecules present in the individual (e.g., the components *p*_1_*H*_2_*, p*_2_*H*_*n*_*, p*_*n*_*H*_1_, not considered here for the sake of simplicity in illustration, but fundamental to the tensor concept) (Figure 6). These mHA-HLA complexes have corresponding T cell receptors *TCR*_1_*, TCR*_2_… *TCR*_*m*_ with affinities, *Z*_1_*, Z*_2_… *Z*_*m*_, the tensor *HpT* may be written as follows,

$$\begin{array}{l} \begin{array}{c} B_{1}Z_{1}\;B_{1}Z_{2}\;B_{1}Z_{m}\\ B_{2}Z_{1}\;B_{2}Z_{2}\;B_{2}Z_{m}\\ B_{n}Z_{1}\;B_{n}Z_{2}\;B_{n}Z_{m} \end{array}\;=\;\begin{array}{c} 1\;0\;0\\ 0\;1\;0\\ 0\;0\;1 \end{array} \end{array}$$

Here *n* and *m* are indices which indicate the HLA-peptide affinity (*Bi*) and TCR binding affinity to the HLA-peptide complex (*Zj*). This is the *alloreactivity tensor*, and it reflects the interaction of the alloreactive peptides with the HLA molecules in that individual and transforms the T cell clonal vector comprised of the array of the T cell clones bearing the above TCR <*T*_*m*_> according to the logistic function.

$$\begin{array}{l} \begin{array}{c} T_{1}^{\prime }\\ T_{2}^{\prime }\\ T_{m}^{\prime } \end{array}=\;\frac{d}{dt}L\;\left(\begin{array}{c} B_{1}Z_{1}\;B_{1}Z_{2}\;B_{1}Z_{m}\\ B_{2}Z_{1}\;B_{2}Z_{2}\;B_{2}Z_{m}\\ B_{n}Z_{1}\;B_{n}Z_{2}\;B_{n}Z_{m} \end{array}\right)\;*\begin{array}{c} T_{1}\\ T_{2}\\ T_{m} \end{array} \end{array}$$

This results in the transformation of the infused donor T cell repertoire, with *T*_1_*, T*_2_… *T*_*m*_ being transformed to *T*_1_'*, T*_2_'… *T*_*m*_' following transplant. The logistic growth equation provides the rule for transformation, so Equation (1) may also be rewritten as follows for the *ith* HLA-bound-peptide, *p*_*i*_, and the responding *jth* T cell clone <*T*_*j*_> in a repertoire comprised of T cell clones *T*_1_ thru *T*_*m*_.

$$\begin{array}{l} <N_{t\;\left(T_{j}\right)}>\;=\;\frac{{<P_{i}c.K_{\left(T_{j}\right)}}^{B_{i}Z_{j}}>*<N_{0\;\left(T_{j}\right)}>}{\left({<P_{i}c.K_{\left(T_{j}\right)}}^{B_{i}Z_{j}}>-\;<N_{t-1\;\left(T_{j}\right)}>\right)\left(e^{-rt<B_{i}>}\right)+1} \end{array}$$

Substituting the value of *r* from Equation (9) in this equation, we get,

(10) $$\begin{array}{l} <\;N_{t\;(T_{j})}\;>\\ \;=\;\frac{<\;P_{i}c.{K_{\left(T_{j}\right)}}^{B_{i}Z_{j}}\;>*<\;N_{0\;(T_{j})}\;>}{\left(<\;P_{i}c.{K_{\left(T_{j}\right)}}^{B_{i}Z_{j}}\;>\;-\;<\;N_{t-1\;(T_{j})}\;>\right)(e^{-\left(CP(S2^{*}Ck\right)t<\;B_{i}\;>})+1}\ldots \end{array}$$

The aggregate alloreactive T cell response at time, *t* then is

$$\begin{array}{l} N_{t\;\left(1\to T_{m}\right)}=\;\overset{m}{\underset{1}{\sum }}<N_{t\;\left(T_{j}\right)}\;> \end{array}$$

This general equation describes the transforming effect of the alloreactivity tensor and the cytokine tensor on the T cell repertoire following SCT. The risk of alloreactivity developing clinically will in this instance be proportional to *N*_*t*(1 → *Tm*)_.

### Dynamical system model of alloreactive T cell response and clinical observations

Does this model explain observations in clinical transplantation? To determine this one may consider the general problem of HLA mismatched SCT and associated negative clinical outcomes (62, 63). In the dynamical system model this phenomenon may be easily understood; the mismatched HLA epitopes are highly expressed so instead of having a low concentration alloreactive protein (the term *P.c* in Equation 2) governing T cell clonal growth, T cell clones bearing TCR that recognize epitopes on the mismatched HLA molecules may encounter an order of magnitude higher target concentration with marked amplification of the steady state alloreactive T cell clonal populations. Indeed, polymorphisms impacting the level of HLA expression correlate with the likelihood of GVHD developing (64). Further any other peptides bound to the mismatched HLA will be novel antigen complexes, to which thymic tolerance and negative selection would not have occurred in the donor, so donor-derived T cell clones will recognize these non-self-antigens and proliferate. This would result in a strong aggregate immune response to the mismatched HLA (and its presented peptides), and this response may be significantly larger than a mHA-HLA directed immune response in the HLA matched setting (65).

Despite the ability of this model to explain some common clinical observations (logistic growth of T cells, power law distributions, and CD4/CD8 clonal distribution), it will not be validated unless it explains the random occurrence of GVHD following allografting. A discussion of this has previously been presented (22), where the competition between non-alloreactive and alloreactive peptides for HLA binding and presentation was invoked as a possible reason for this, resulting in a probability distribution (ρ*Hp*_*n*_) for the alloreactive peptide *p*_*n*_ to be presented on HLA molecule *H*. A further consideration in the development of GVHD from these alloreactive T cell clonal growth simulations is the probability function introduced by peptide cleavage potential, which affects the likelihood of antigen presentation, as well as whether the relevant T cell clones are present following transplantation (ρ*T*_*m*_). The probability of peptide cleavage (ρ*p*_*cl*_) is determined by the amino-acid sequence at the C terminal of the peptide antigens (66), as such, several peptides in our study may have low likelihood of presentation and may be ignored to simplify the model. The likelihood of alloreactive antigen response (ρ_*GVHD*_) may then be calculated as

(11) $$\begin{array}{l} \rho_{GVHD}=\left(\rho_{p_{cl}}*\rho_{Hp_{n}}\right)*\rho_{T_{m}}\ldots . \end{array}$$

Computed for each alloreactive peptide, the probability of clonal expansion of the mHA-targeting-T cells will be significantly diminished as the number of probability terms are introduced into the computations, which explains why despite many potential alloreactive antigens being present in each donor and recipient not every patient develops GVHD.

Another clinical phenomenon, the T cell growth amplification effect of cytokines is well recognized clinically. This is recognized in both the need for lymphodepletion prior to adaptive immunotherapy and in the cytokine release syndrome seen following it (67, 68). Thus far in the dynamical system model discussed above the cytokine tensor effect has been described as modulating rate of T cell clonal growth. However, cytokines effect not only the rate, but they also effect the magnitude of clonal expansion, amplifying the T cell clonal growth. This may be modeled using the iterating equation

(12) $$\begin{array}{l} N_{t\;(T_{x})}=\left(Ck^{t(1-(N_{t(Tx)}/K_{\left(Tx\right)})}\right).\\ \left(\frac{(P_{y}.c.{K_{\left(T_{x}\right)}}^{B_{y}Z_{x}})*N_{0(T_{x})}\;}{\left((P_{y}.c.{K_{\left(T_{x}\right)}}^{B_{y}Z_{x}})-N_{t-1(T_{x})}\right)(e^{-\left(CP(S2*Ck\right)tB_{y}})+1}\right)\ldots \end{array}$$

This equation demonstrates the effect of the cytokine tensor, *Ck*, as a time-dependent function, which in the beginning increases the magnitude of T cell clonal growth for clones expressing the relevant cytokine receptors by an order of magnitude. As the number of T cells increases, this time-dependent effect declines to a steady state level since the cytokines are taken up and utilized by the growing T cell population. This relationship plotted over time demonstrates the familiar T cell antigen response curve and mirrors the effect of antigen presenting cell growth previously described (Supplementary Figure 4) [See Koparde et al (22), for discussion of APC-T cell interactions].

A final consideration in building this model is that the antigen matrices presented above are “identity matrices” with binary values of, 1 along the diagonal of a square matrix and 0 elsewhere. In physiologic conditions, however there will be a continuum of values because of differential binding of peptides to various HLA molecules and cross reactivity of T cell receptors with such antigen complexes, generating *random number matrices*, rather than identity matrices (69). This will add another element of complexity to the antigen-effector interactions, and possibly provides a rationale for complex GVHD phenotypes observed.

In conclusion, the considerable genetic variation present between HLA matched transplant donors and recipients, when analyzed *in silico*, yields a putative large array of recipient mHA bound to both HLA class I and class II molecules (Supplementary Table 3). The peptide-HLA antigen findings reported here are likely to be revised and refined in the future as the computational biology approach becomes more sophisticated to account for the variables not considered in this analysis. However, the value of this work lies in the mathematical principles of T cell response to antigen arrays it helps illustrate. This mathematical model may be used to optimize donor selection and titrate immunosuppression, first, by utilizing exome sequencing to determine the alloreactive antigen profile. Second, contemporary high throughput sequencing of T cell receptors would allow identification of antigen-HLA specific motifs (70); extended to the donor T cells this may be used to identify potential alloreactive T cell clones (37, 71). These insights using a combination of next generation sequencing and mathematical models accounting for the complexity of alloreactive immune responses may be utilized to allow greater precision in stem cell donor selection and management of immunosuppression following transplantation in particular and cancer immunotherapy in general.

## Ethics statement

The Virginia Commonwealth University's Institutional Review Board approved this study. Previously cryopreserved DNA samples, de-identified by clinical research staff, were used for this study. The samples were acquired from archived DNA remaining after post-transplant studies in the VCU Molecular Biology laboratory. The VCU IRB waived the need for informed consent on all adult participants as samples were all archived and previously de-identified with only VCU BMT research staff and the study PI retaining access to any PHI involved in retrospective analysis. The Sequencing team did not have access to the sample identity and clinical team did not have access to exome sequencing data.

## Author contributions

VK performed bioinformatic analysis of the sequencing data to identify unique peptides and their HLA binding affinity, as well as tissue expression and wrote the paper. MS performed sequencing on samples identified and procured by CR. AS analyzed the data, performed statistical analysis and wrote the paper. AS, CH, and MJ-L created data files with unique peptides and HLA IC50 values and wrote the paper. AT, MN, and GB developed the WES study. AT developed the vector-operator and tensor models and wrote the manuscript. JR, RQ, MN, SH, DW, BA, and JM critically reviewed the manuscript and provided expert commentary. All the authors contributed to writing the manuscript. Sequencing and Bioinformatics Analysis was performed in the Genomics Core of the Nucleic Acids Research Facilities at VCU, supervised by GB.

### Conflict of interest statement

The authors declare that the research was conducted in the absence of any commercial or financial relationships that could be construed as a potential conflict of interest.
