# Supplementary material for: Determining the Quantitative Principles of T Cell Response to Antigenic Disparity in Stem Cell Transplantation
Source: Front Immunol. 2018 Oct 11;9:2284. doi: 10.3389/fimmu.2018.02284 (PMC6193078; doi:10.3389/fimmu.2018.02284)
Supplement: Supplementary file 1 [file Table_1.DOCX]

**Supplementary Table 1.** Demographics
