# Supplementary material for: Determining the Quantitative Principles of T Cell Response to Antigenic Disparity in Stem Cell Transplantation
Source: Front Immunol. 2018 Oct 11;9:2284. doi: 10.3389/fimmu.2018.02284 (PMC6193078; doi:10.3389/fimmu.2018.02284)
Supplement: Supplementary file 2 [file Table_2.DOCX]

**Supplementary Table 2.** Explanation of variables used in the equations.

| **Variable** | **Description** | **Previously** |
| --- | --- | --- |
| *T* | T cell clone |  |
| *N* | T cell count at various times (time indicated by subscripts) |  |
| *K* | Proliferation constant determining steady state T cell count |  |
| *t* | time/iterations |  |
| *r* | Growth rate |  |
| *e* | 2.7182, base of natural logarithms |  |
| *p* | Alloreactive peptide |  |
| *H* | HLA molecules |  |
| *TCR* | T cell receptor of T cell clone, T |  |
| *B* | Binding affinity of peptide, *p* for HLA molecule, *H* | *afm*HA ^23^ |
| *Z* | Binding affinity TCR for mHA-HLA complex (*Hp*) | a*f*TCR ^23^ |
| *P* | Tissue expression of protein with alloreactive peptide *p* | *P_exp_*^23^ |
| *c* | Constant which reduces *P* for HLA class II presentation |  |
| *HpT* | Alloreactivity tensor |  |
| *Ck* | Cytokine tensor |  |
| *CP* | Checkpoint molecule |  |
| *S2* | Signal 2 |  |
| *L* | Logistic operator |  |
| *ρ* | Probability |  |
