# Supplementary material for: Determining the Quantitative Principles of T Cell Response to Antigenic Disparity in Stem Cell Transplantation
Source: Front Immunol. 2018 Oct 11;9:2284. doi: 10.3389/fimmu.2018.02284 (PMC6193078; doi:10.3389/fimmu.2018.02284)
Supplement: Supplementary file 3 [file Table_3.DOCX]

**Appendix**

*Demographic factors influencing HLA class II bound alloreactive peptides*

Finally, demographic factors, including race and gender, that impact genetic disparity were analyzed. African-American vs. Caucasian DRP demonstrated a larger number of HLA-DRB1 bound mHA in African American DRP, for both BP (74,179 vs. 53,735 in Caucasian patients; p=0.075) and SB peptides (11,972 vs. 7,503; p=0.36). There was no significant difference in the number of BP or SB in the gender-matched male or female DRP, not accounting for Y chromosome disparity in male patients receiving transplant from a female donor.
