# Supplementary figures and images for: Determining the Quantitative Principles of T Cell Response to Antigenic Disparity in Stem Cell Transplantation
Source: Front Immunol. 2018 Oct 11;9:2284. doi: 10.3389/fimmu.2018.02284 (PMC6193078; doi:10.3389/fimmu.2018.02284)

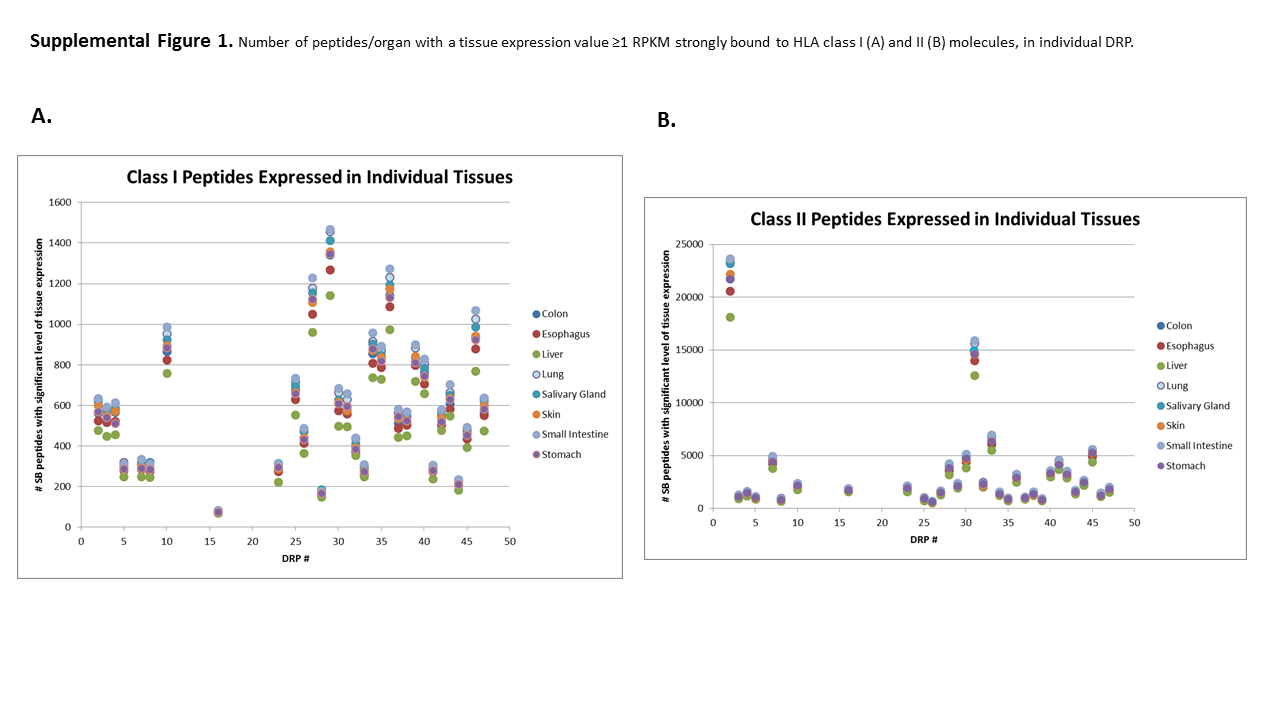

Supplement: Supplementary file 4 [file Image_1.tif]

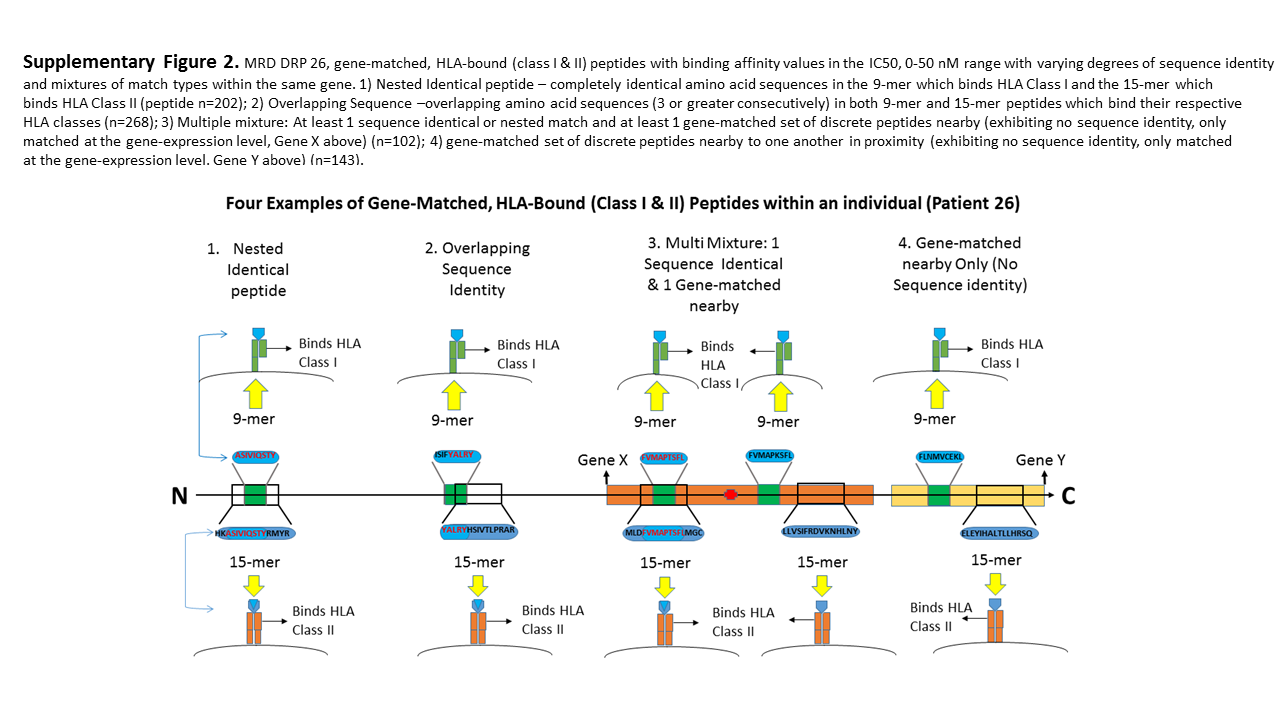

Supplement: Supplementary file 5 [file Image_2.tif]

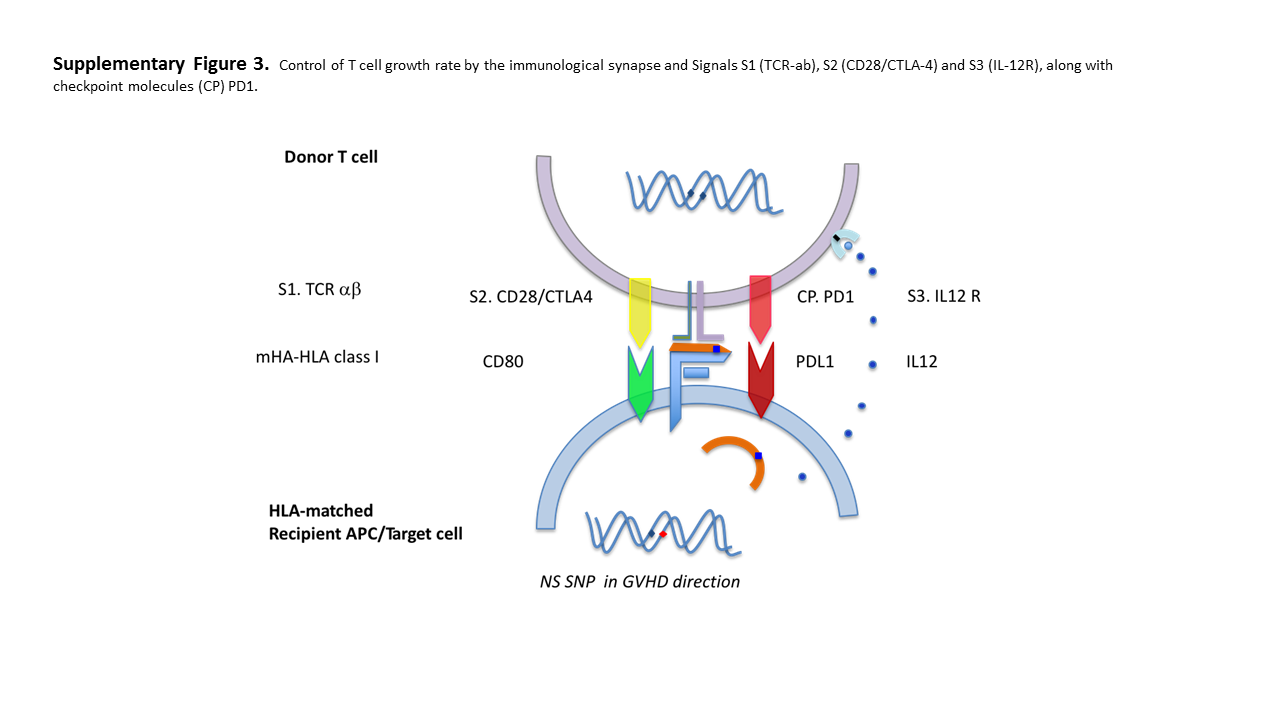

Supplement: Supplementary file 6 [file Image_3.tif]

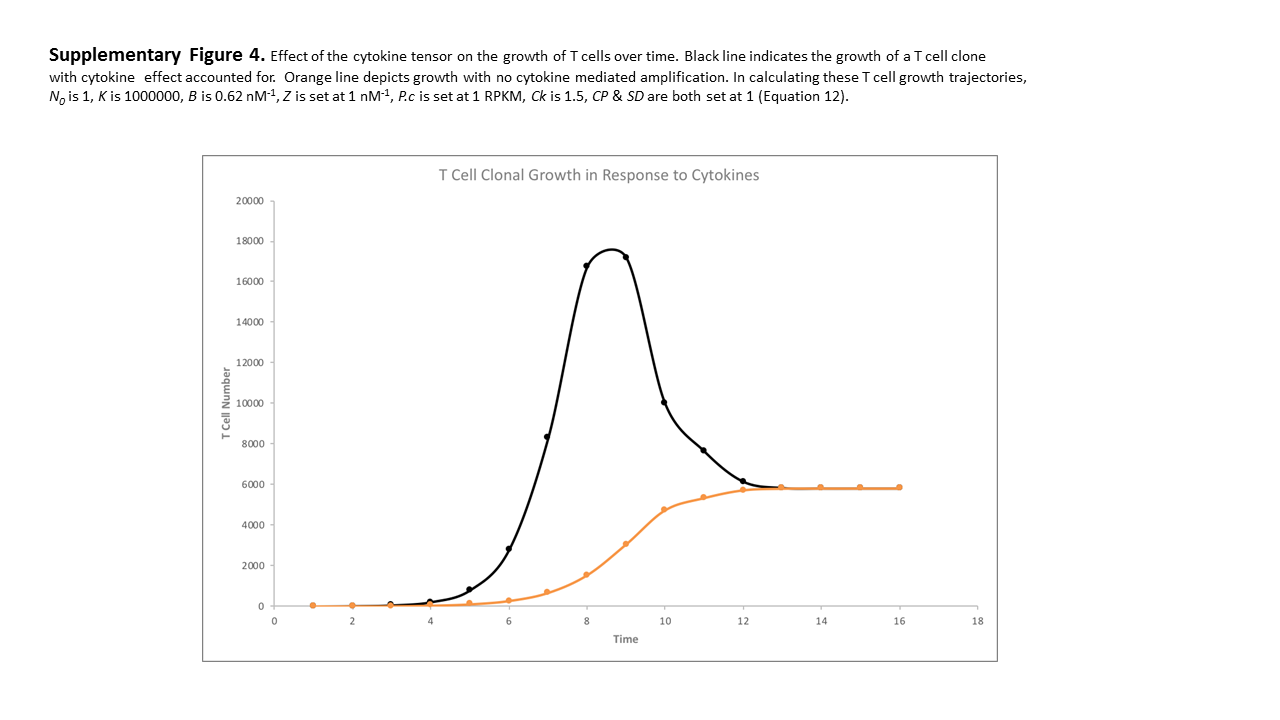

Supplement: Supplementary file 7 [file Image_4.tif]
